# A Pilot Study for Metabolic Profiling of Obesity-Associated Microbial Gut Dysbiosis in Male Wistar Rats

**DOI:** 10.3390/biom11020303

**Published:** 2021-02-18

**Authors:** Julia Hernandez-Baixauli, Pere Puigbò, Helena Torrell, Hector Palacios-Jordan, Vicent J. Ribas Ripoll, Antoni Caimari, Josep M Del Bas, Laura Baselga-Escudero, Miquel Mulero

**Affiliations:** 1 Eurecat, Centre Tecnològic de Catalunya, Unitat de Nutrició i Salut, 43204 Reus, Spain; julia.hernandez@eurecat.org (J.H.-B.); pere.puigbo@eurecat.org (P.P.); antoni.caimari@eurecat.org (A.C.); laura.baselga@eurecat.org (L.B.-E.); 2 Department of Biochemistry and Biotechnology, Universitat Rovira i Virgili, 43007 Tarragona, Spain; 3 Department of Biology, University of Turku, 20014 Turku, Finland; 4 Eurecat, Centre Tecnològic de Catalunya, Centre for Omic Sciences (COS), Joint Unit Universitat Rovira i Virgili−EURECAT, 43204 Reus, Spain; helena.torrell@eurecat.org (H.T.); hector.palacios@eurecat.org (H.P.-J.); 5 Eurecat, Centre Tecnològic de Catalunya, eHealth Unit, 08005 Barcelona, Spain; vicent.ribas@eurecat.org; 6 Nutrigenomics Research Group, Department of Biochemistry and Biotechnology, Universitat Rovira i Virgili, 43007 Tarragona, Spain

**Keywords:** microbial dysbiosis, gut microbiota, metagenomics, metabolomics, dysbiosis biomarkers, metabolic profile, diacylglycerol 34:2, hippurate, 3-HPPA, *o*-coumaric acid

## Abstract

Obesity is one of the most incident and concerning disease worldwide. Definite strategies to prevent obesity and related complications remain elusive. Among the risk factors of the onset of obesity, gut microbiota might play an important role in the pathogenesis of the disease, and it has received extensive attention because it affects the host metabolism. In this study, we aimed to define a metabolic profile of the segregated obesity-associated gut dysbiosis risk factor. The study of the metabolome, in an obesity-associated gut dysbiosis model, provides a relevant way for the discrimination on the different biomarkers in the obesity onset. Thus, we developed a model of this obesity risk factors through the transference of gut microbiota from obese to non-obese male Wistar rats and performed a subsequent metabolic analysis in the receptor rats. Our results showed alterations in the lipid metabolism in plasma and in the phenylalanine metabolism in urine. In consequence, we have identified metabolic changes characterized by: (1) an increase in DG:34:2 in plasma, a decrease in hippurate, (2) an increase in 3-HPPA, and (3) an increase in *o*-coumaric acid. Hereby, we propose these metabolites as a metabolic profile associated to a segregated dysbiosis state related to obesity disease.

## 1. Introduction

Obesity has been defined as an excessive or abnormal accumulation of fat that represents a significant health risk [1]. The dramatical increase in the incidence of obesity worldwide, during the last 20 years across all ages, has changed the health perspective regarding this condition [2]. In fact, the term has evolved to “globesity,” referring to the acquired pandemic characteristic of this condition due to globalization [3].

Lots of efforts have been devoted to try to decrease the incidence and prevalence, as well as the health complications associated to obesity [4]. For instance, obesity-associated risk factors have been associated with a large number of chronic diseases, including cardiovascular diseases (e.g., heart disease or stroke), which are the leading causes of death worldwide [5]. Furthermore, being obese can also lead to important disorders, including diabetes and its associated conditions [6] and musculoskeletal disorders, such as osteoarthritis [7]. In accordance to that, since 1980, the rates of diabetes have quadrupled around the world [8]. Finally, even some cancers (including endometrial, breast, ovarian, prostate, liver, gallbladder, kidney, and colon cancers) have also been associated with obesity [9,10]. Interestingly, the risk of these noncommunicable diseases significantly increases even when a person is only slightly overweight and grows more seriously as the body mass index (BMI) rises [11,12].

Unfortunately, definite strategies to tackle the prevention of obesity, and its related complications, remain elusive. In this regard, epidemiological studies have highlighted some potential environmental exposures, including diet, energy expenditure, early life influences, sleep deprivation, endocrine disruptors, chronic inflammation, and altered gut microbiota (GM) status, as important contributors to a higher obesity risk [13,14,15]. Among these, the GM has received extensive attention during the previous decade because it has been shown that manipulation of the GM may affect the host metabolism. In this sense, it has been proved that obesity is accompanied by a deep alteration of the host microbiota, and such condition has been defined as intestinal or gut dysbiosis [16,17,18].

In consequence, it has been demonstrated that the variation in GM might play an important role in the pathogenesis of obesity [19]. Although in healthy individuals the composition of intestinal microbiota is highly diverse, those exhibiting obesity, insulin resistance, and dyslipidemia are characterized by low bacterial richness [20]. Moreover, the GM composition differs between obese and lean individuals [20], e.g., *Bacteroidetes* abundance is lower in obese individuals [20], and this proportion increases along with weight loss based on a low-calorie diet [21]. *Lactobacillus* and *Clostridium* spp. are associated with insulin resistance, being *Lactobacillus* positively correlated with fasting glucose and HbA1c levels, whereas *Clostridium* showed a negative correlation with these parameters [22,23]. These data suggest that specific bacteria, as well as certain microbial metabolic activities, could be beneficial or detrimental to the onset of obesity. Therefore, the GM has been suggested to be a driving force in the pathogenesis of obesity [24].

Although the evidence for many classical obesity biomarkers (i.e., adiponectin and C-reactive protein) has been initially promising in disease etiology, the evidence for a clear causal role in humans remains limited [25]. Furthermore, the ability to improve disease prediction has been little demonstrated beyond classical biomarkers. Hereby, it is time to focus on the risk factors of the onset of obesity to open to novel biomarkers discerning between health and disease. Consequently, in the “precision medicine” era, there is an increasing demand of novel and growing sources of potentially promising biomarkers, such as adipokines, cytokines, metabolites, and microRNAs, which are related to obesity and could bring new improvement to personalized prevention [26]. The field of metabolomics has been increasing as an important tool for the prognosis, and diagnosis, of different diseases stages, by investigating the endogenous levels of small metabolites in clinical practice from different biofluids standing out plasma/serum and urine [27,28]. Furthermore, the scientific community has been called to use these tools to obtain information about the metabolism and potential biomarkers of obesity-associated risk factors [29].

Importantly, reshaping the GM has been shown as an effective strategy in weight loss and metabolic diseases amelioration [30]. To illustrate this fact, in a recent study with obese participants for avoiding weight gain after a weight reduction treatment, an autologous fecal microbiota transplantation was proposed to prevent weight regain (instead to modify the diet). The experimental approach focused on the idea that microbiota is more important to modulate obesity than diet [31]. Interestingly, the results showed that the autologous fecal microbiota transplantation preserved weight loss, and it was useful for glycemic control [31].

However, the gradual changes in the GM during weight gain and the related onset of metabolic abnormalities is still unclear in obesity [32]. In this sense, due to the urgent need of development of new and more effective strategies for disease prevention, a better understanding of the obesity pathophysiology, as well as new obesity-related biomarkers, are constantly demanded. In consequence, as GM plays such important role in obesity, a myriad of GM obesity associated biomarkers has been discovered. For example, it has been shown that the size and composition of bile acid pool can change due to GM’s alterations, and this may evolve with subsequent altered signaling and activation of bile acid receptors such as farnesoid X receptor (FXR) and Takeda G protein-coupled receptor 5 (TGR5) and perturb, in consequence, lipid and glucose homeostasis [33,34]. Moreover, dysbiosis alters short-chain fatty acids (SCFA) production with a consequent altered secretion of gut peptide YY(PYY) and glucagon-like peptide 1 (GLP-1), thus affecting appetite and satiety [35,36]. Similarly, biomarkers for GM metabolites and by-products may increase gut permeability and nutrient absorption and therefore, additionally contribute to obesity. On the other hand, the main factor responsible for inducing increased gut permeability and microbiota translocation into host interior is the bacterial by-product lipopolysaccharide. Nevertheless, due to the diverse outcomes of obesity-related complications (e.g., insulin resistance, inflammation, or gut dysbiosis), that are intimately related to each other, there is a lack of knowledge about the segregated effect of each obesity-related complications on metabolism and its specific biomarkers.

In this study, we have stablished a pilot study for metabolic profiling of obesity-associated microbial gut dysbiosis. In this sense, the transference of GM from obese to non-obese rats could allow us the discovery of novel discriminatory biomarkers related to this GM alterations, providing valuable information about the origin of obesity-associated biomarkers.

## 2. Materials and Methods

### 2.1. Animal Procedure

The Animal Ethics Committee of the Universitat Rovira i Virgili (Tarragona, Spain) approved all the procedures (code 10454). The experimental protocol followed the “Principles of Laboratory Care” and was carried out in accordance with the European Communities Council Directive (86/609/EEC). All animals were housed individually at 22 °C with a light/dark cycle of 12 h (lights on at 9 a.m.) and were given access to food and water ad libitum during all the experiment. Individual housing allows to determine an accurate estimation of food intake and to avoid crossed effects on microbiota because of the “coprophagy effect” usually shown in rats. Animals were randomly assigned to the different groups considering similar average body weight. Body weight and food intake were recorded weekly. For food intake estimation, the chow weight was assessed before and after 24 h of the consumption. 

**Figure 1:**
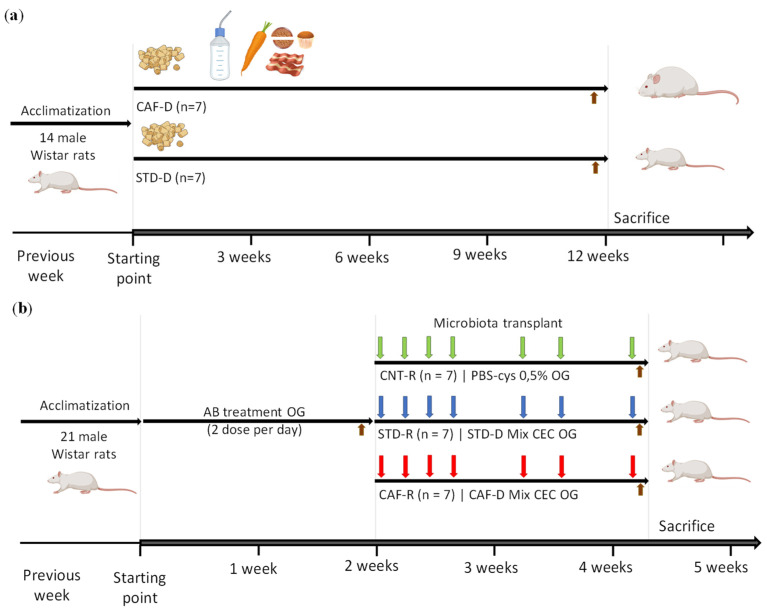
Schematic representation of the experimental design. (**a**) Obtention of cecum (CEC) donors induced by a cafeteria diet (CAF-D) and a standard diet (STD-D) (**b**) Obesity-associated gut dysbiosis with a healthy phenotype induced by cecal donors transplantation, including the previous depletion of host microbiota and the cecal content transplantation in healthy population. Brown arrow, feces (FCS) collection; green arrow, control group (CNT-R); blue arrow, standard receptors (STD-R); red arrow, cafeteria receptors (CAF-R). OG, oral gavage; AB, antibiotic.

The whole study was planned in two differentiated steps: in the first experimental part, the CEC of cafeteria diet donors (CAF-D) and standard diet donors (STD-D) groups were obtained to collect the cecal content, and in the second experimental part, healthy rats corresponding to the cafeteria receptors (CAF-R) and the standard receptors (STD-R) groups received the cecal content of the donors, including a control group receiving the vehicle (CNT-R) (Figure 1).

#### 2.1.1. Obtention of Cecal Donors Induced by Cafeteria Diet and Standard Diet

The first part of the experiment was performed using 14 male 8-week-old Wistar rats (Harlan Laboratories, Barcelona, Spain), which were randomly distributed into two experimental groups (n = 7). Afterwards, they were fed with two different diets depending on the group for 12 weeks (Figure 1a): the animals from the STD-D group were fed with standard chow diet (Tecklad Global 18% Protein Rodent Diet 2014, Harlan, Barcelona, Spain), and the animals from the CAF-D group were fed with a cafeteria diet with the following components (quantity per rat): bacon (8–12 g), biscuit with pâté (12–15 g), biscuit with cheese (10–12 g), muffins (pastry) (8–10 g), carrots (6–8 g), milk with sugar (220 g/L; 50 mL), water (ad libitum), and also with standard chow. Sample size and nutrient compositions of CAF-D and STD-D used herein have been previously described in the literature [37]. The day before the sacrifice, feces (FCS) were collected to perform metagenomics. The animals were killed after 7 h of fasting by guillotine under anesthesia (pentobarbital sodium, 50 mg/kg per body weight), and cecum (CEC) was rapidly removed, weighed, frozen in liquid nitrogen, and stored at −80 °C for GM preservation. For the preparation of the second part of the experiment, the cecal content of each group was pooled and diluted in 0.5% PBS-cys (4 g of CEC/15 mL of 0.5% PBS-cys). The cecal mixture was centrifuged to eliminate solid residues and to facilitate the subsequent administration by oral gavage. Finally, the mixture of each group was aliquoted (single-dose of 1.1 mL) and stored at −80 °C for further treatment.

#### 2.1.2. Model of Obesity-Associated Gut Dysbiosis with a Healthy Phenotype Induced by Cecal Transplantation 

The second part of the experiment was carried out using 21 8-week-old male Wistar rats (Harlan Laboratories, Barcelona, Spain) and the procedure consisted into two parts (Figure 1b). First, all the rats were treated with an antibiotic cocktail to deplete the host microbiota. After the antibiotic treatment, the rats were randomly distributed into three experimental groups (n = 7) for the restoration of the microbiota by the administration of the vehicle or the external GM (CNT-R, STD-R, and CAF-R groups). The animals were fed with a standard chow diet ad libitum (Teklad Global 18% Protein Rodent Diet 2014, Harlan, Barcelona, Spain). All the procedure was carried out with the maximum sterility.

The antibiotic cocktail was administered by oral gavage twice daily for 13 consecutive days to all groups (at 10:00 a.m. and 5:00 p.m.). It included a mixture of vancomycin (50 mg/kg), neomycin, and metronidazole (each at 100 mg/kg). In addition, the drinking water was supplemented with ampicillin (1 g/L) during the antibiotic treatment to avoid the growing of microorganism during the treatment [38]. At the end of the antibiotic treatment, FCS were collected to check the depletion of the host microbiota.

All animals received omeprazole (20 mg/kg) by oral gavage 24 h after the last antibiotic treatment and 4–5 h before every transplant to reduce the acidification of the environment and to allow the survival of microorganisms through the gastrointestinal tract. All the treatments were administered by oral gavage, and they consisted of following treatments: the CNT group was treated with 0.5% PBS-cys and the STD-R and CAF-R groups with STD-D and CAF-D cecal mix prepared in the first experiment, respectively. The microbiota transplant consisted of 4 consecutive days of treatment during the first week, 2 reminders on the second week, and finally, a weekly reminder over the last 2 weeks with 4 weeks of total duration. The day before the sacrifice, FCS and urine were collected to perform metagenomics and metabolomics, respectively. Urine was collected following the recommended hydrophobic sand method (avoiding stress and metabolic changes) [39]. For each rat, a single 300 g pack of hydrophobic sand was spread (LabSand, Coastline Global, Palo Alto, CA, USA) on the bottom of a mouse plastic microisolation cage. Urine was gathered with sodium azide (Sigma, St Louis, MO, USA) as preservative every half hour for 6 h and was subsequently pooled at the end of the session. The FCS and pooled urine samples for each animal were stored at − 80 °C until further analysis. At the end of the study, rats were killed under anesthesia (pentobarbital sodium, 50 mg kg^−1^ body weight) by guillotine after 7 h of fasting to avoid interferences of the early postprandial state in plasma metabolites. Blood was collected, and plasma was obtained by centrifugation (2000× *g* for 15 min at 4 °C) and stored at −80 °C until analysis. Tissues were rapidly removed, weighted, snap-frozen in liquid nitrogen, and stored at −80 °C until further analyses.

### 2.2. Biochemical Parameters

#### 2.2.1. Plasma Parameters

Enzymatic colorimetric kits were used for the determination of plasma total cholesterol, triglycerides, and glucose (QCA, Barcelona, Spain) and non-esterified free fatty acids (NEFAs) (WAKO, Neuss, Germany).

#### 2.2.2. Liver Lipid Parameters

Liver lipids were extracted and quantified from a 100–120 mg liver piece using a method previously described in the literature [40]. Briefly, lipids were extracted with 1 mL of hexane/isopropanol (3:2, *v*/*v*) and degassed with gas nitrogen before leaving overnight under orbital agitation at room temperature protected from light. After an extraction with 0.3 mL of Na_2_SO_4_ (0.47 M), the lipid phase was dried with nitrogen gas and total lipids were quantified gravimetrically before emulsifying as described previously [41]. Triglycerides, cholesterol, and phospholipids were measured with commercial enzymatic kits (QCA, Barcelona, Spain).

### 2.3. Metagenomic Analysis

The genomic bacterial DNA was obtained from 700 to 1000 mg of FCS and CEC of previously collected with the QIAamp DNA stool kit (Qiagen, Hilden, Germany; cat. no. 51504) following the manufacturer’s protocol. Partial 16S ribosomal RNA gene sequences were amplified from 20 ng of extracted DNA using three primer pairs, which target the V3, V4, and V6 regions, respectively. Equimolar pools of each fragment were combined to create the DNA library, which was subjected to a clonal amplification by an emulsion PCR. After an Ion Sphere Particle enrichment process, samples were loaded onto 318 chips and sequenced using the Ion Torrent PGM (Life Technologies, Carlsbad, CA, USA). The individual sequence reads were filtered by the PGM software (Life Technologies, Carlsbad, CA, USA) to remove low-quality and polyclonal sequences. Those reads were processed using QIIME [42], selecting only sequences with 150–200 bp and omitting homopolymers. 16S ribosomal RNA operational taxonomic units (OTUs) were assigned using uclust (>97% sequence homology) and a reference data set from Greengenes (Lawrence Berkeley National Laboratory, Berkeley, CA, USA).

### 2.4. Metabolomic Analysis: Plasma and Urine Approach

The method for the extraction of plasma lipids was ultrahigh performance liquid chromatography coupled with quadrupole time-of-flight (UHPLC-qTOF). For the extraction of the hydrophobic lipids, a liquid–liquid extraction based on the Folch procedure was performed by adding four volumes of chloroform:methanol (2:1, *v*/*v*) containing internal standard mixture (Lipidomic SPLASH^®^) to plasma. Then, the samples were mixed and incubated at −20 °C for 30 min. Afterwards, water with NaCl (0.8%) was added, and the mixture was centrifuged at 21.420× *g*. Lower phase was recovered, evaporated to dryness, reconstituted with methanol:methyl-tert-butyl ether (9:1, *v*/*v*), and analyzed by UHPLC-qTOF (model 6550 of Agilent, Santa Clara, CA, USA) in positive electrospray ionization mode. The chromatographic consists in an elution with a ternary mobile phase containing water, methanol, and 2-propanol with 10 mM ammonium formate and 0.1% formic acid. The stationary phase was a C18 column (Kinetex EVO C18 Column, 2.6 µm, 2.1 mm × 100 mm) that allows the sequential elution of the more hydrophobic lipids such as TG, diacylglycerols (DG), phosphatidylcholines (PC), cholesterol esters (ChoE), lysophospholipids (LPC), and sphingomyelins (SM), among others. The identification of lipid species was performed by matching their accurate mass and tandem mass spectrum, when available, to Metlin-PCDL from Agilent containing more than 40,000 metabolites and lipids. In addition, chromatographic behavior of pure standards for each family and bibliographic information was used to ensure their putative identification. After putative identification of lipids, these were semiquantified in terms of internal standard response ratio using one internal standard for each lipid family.

The methodology followed for the extraction of plasma metabolites was gas chromatography coupled with quadrupole time-of-flight (GC-qTOF). For the extraction, a protein precipitation extraction was performed by adding eight volumes of methanol:water (8:2, *v*/*v*) containing internal standard mixture (succinic acid-d4, myristic acid-d27, glicerol-^13^C3, and D-glucose-^13^C6) to plasma samples. Then, the samples were mixed and incubated at 4 °C for 10 min and centrifuged at 21.420× *g*, and supernatant was evaporated to dryness before compound derivatization (metoximation and silylation). The derivatized compounds were analyzed by GC-qTOF (model 7200 of Agilent, Santa Clara, CA, USA). The chromatographic separation was based on the Fiehn method, using a J&W Scientific HP5-MS (30 m × 0.25 mm i.d.), 0.25 µm film capillary column, and helium as carrier gas using an oven program from 60 to 325 °C. Ionization was done by electronic impact (EI), with electron energy of 70 eV and operated in full scan mode. The identification of metabolites was performed by matching their EI mass spectrum and retention time to metabolomic Fiehn library (Agilent, Santa Clara, CA, USA), which contains more than 1400 metabolites. After putative identification of metabolites, these were semiquantified in terms of internal standard response ratio.

The methodology followed for the extraction of urine metabolites was proton nuclear magnetic resonance (^1^H-NMR). The urine sample was mixed (1:1, *v*/*v*) with phosphate buffered saline containing with 3-(Trimethylsilyl)propionic-2,2,3,3-d4 acid sodium salt (TSP) (Sigma Aldrich) and placed on a 5 nm NMR tube for direct analysis by ^1^H-NMR. ^1^H-NMR spectra were recorded at 300 K on an Avance III 600 spectrometer (Bruker^®^, Karlsruhe, Germany) operating at a proton frequency of 600.20 MHz using a 5 mm PBBO gradient probe. Diluted urine aqueous samples were measured and recorded in procno 11 using a one-dimensional ^1^H pulse. Experiments were carried out using the nuclear Overhauser effect spectroscopy (NOESY). NOESY presaturation sequence (RD–90–t1–90–tm–90 ACQ) was used to suppress the residual water peak, and the mixing time was set at 100 ms. Solvent presaturation with irradiation power of 150 μW was applied during recycling delay (RD = 5 s) and mixing time (noesypr1d pulse program in Bruker^®^) to eliminate the residual water. The 90-pulse length was calibrated for each sample and varied from 11.21 to 11.38 ms. The spectral width was 9.6 kHz (16 ppm), and a total of 128 transients were collected into 64 k data points for each ^1^H spectrum. The exponential line broadening applied before Fourier transformation was of 0.3 Hz. The frequency domain spectra were manually phased and baseline-corrected using TopSpin software (version 3.2, Bruker). Data was normalized by two different ways, by probabilistic method, to avoid differences between sample due to different urine concentration, and by ERETIC software. The acquired 1H-NMR spectra were compared to references of pure compounds from the metabolic profiling AMIX spectra database (Bruker^®^), HMDB, and Chenomx databases for metabolite identification. In addition, we assigned metabolites by ^1^H-^1^H homonuclear correlation (COSY and TOCSY) and ^1^H-^13^C heteronuclear (HSQC) 2D NMR experiments and by correlation with pure compounds run in-house. After pre-processing, specific ^1^H-NMR regions identified in the spectra were integrated using MATLAB scripts run in house. Curated identified regions across the spectra were exported to excel spreadsheet to evaluate robustness of the different ^1^H-NMR signals and to give relative concentrations.

### 2.5. Pathway Analysis

The KEGG (Kyoto Encyclopedia of Genes and Genomes) pathway map was used to interpret the metabolomic data in the context of biological processes, pathways, and networks [43]. The most important features were analyzed through KEGG to elucidate the global effect in metabolism.

### 2.6. Statistical Analysis

The statistical analysis was performed using the R software (version 4.0.1) and different libraries included in Bioconductor (version 3.11). The biochemical data are expressed as the mean ± standard error of the mean (S.E.M.). Parametric unpaired *t*-test after a normality study was used for single statistical comparisons, thus a two-tailed value of *p* < 0.05 was considered. After parametric unpaired *t*-test, *p*-value adjustment for multiple comparisons was performed according the Benjamin-Hochberg (B-H) correction considering a 5% of false discovery rate (FDR). The magnitude of difference between populations was determined by the determination of Fold Change (FC). For metagenomics, the number of OTUs per sample were scaled so each sample had the same mean and were filtered to only include OTUs that were present at 0.1% of the total counts in at least 3 samples [44]. Further, the random forest classifier was calculated to sort the most important metabolites that distinguish between the control (STD-R) and obesity-associated gut dysbiosis (CAF-R) group. Finally, correlation analysis between metagenomics and metabolomics were performed by kernel density plot and the correspondent test of equal densities. 

### 2.7. Limitations

However, this research is limited by several shortcomings. As occurs in some studies, the design of the current study must be considered as a “pilot study” because the number of the animals is not high enough (n = 7 per group) to provide a strong statistical conclusion about the biomarkers. In addition, females must be included for further studies. Nonetheless, these metagenomic results must be interpreted with caution because 16S sequencing was performed. These issues should be considered to perform further experiments.

## 3. Results

### 3.1. Characterization and Metagenomic Analysis of Donor Animals: Induced by Cafeteria Diet and Standard Diet

Animals fed with an obesogenic diet presented a significant increase in body weight (g) (CAF-D = 550.09 ± 18.17 and STD-D = 443.05 ± 24.92; *p* = 0.005) and a huge decrease in CEC weight (g) (CAF-D = 4.34 ± 0.17 and STD-D = 5.59 ± 0.19; *p* < 0.001), respect those fed with a standard diet, in agreement with other researchers [45,46]. 

To study the metabolic alterations of obesity-associated gut dysbiosis with a healthy phenotype, the previous step was the obtention of CEC donors for further transplant. Therefore, a metagenomic analysis was performed in donor groups (CAF-D and STD-D) in CEC and FCS to check the success on the obesity-associated gut dysbiosis. The reads count in 16S rRNA gene sequencing were 200.624–988.148 per sample. 

Results from CEC showed a significant change in the two major phyla of the GM: *Firmicutes* (STD-D: 85.26%, CAF-D: 49.39%; *q* = 0.001) decreased and *Bacteroidetes* (STD-D: 12.83%, CAF-D: 34.77%; *q* = 0.025) increased in CAF-D group. Thus, there was an increase of the ratio of *Bacteroidetes*/*Firmicutes* in the CAF-D group (STD-D = 0.16 ± 0.04 and CAF-D = 0.81 ± 0.21; *p* = 0.009). Nevertheless, these alterations were not observed in FCS. Moreover, the results showed some changes in less represented phyla, e.g., the phylum *Tenericutes* was significantly decreased in the CAF-D group (STD-D: 0.29%, CAF-D: 0.11%; *q* = 0.034), the *Proteobacteria* (STD-D: 0.31%, CAF-D: 3.47%; *q* = 0.034) was increased, and the phylum *Verrucomicrobia* was almost significantly increased (STD-D: 1.21%, CAF-D: 12.01%; *q* = 0.053). 

Focusing on genera, the differences between donor groups were summarized in Table S1 standing out that *Clostridiales* and *Bacteroidales* were the most altered taxa representing the main differences in *Firmicutes* and *Bacteroidetes* phyla, respectively. In the CAF-D group, some genera experienced changes as well: there was a significant increase of *Ruminococcus*, *Blautia*, and *Parabacteroides*. Some differences were common between FCS and CEC at genus level, as both experienced a significant decrease in an uncharacterized genus belonging to of *Clostridiales* (STD-D: 29.73%, CAF-D: 5.59%; *q* = 0.019) and an increase of *Parabacteroides* genus (STD-D: 0.42%, CAF-D: 3.44%; *q* = 0.047) in the CAF-D group. Other genus significantly decreased in CAF-D FCS group were, e.g., two *Clostridiales*, *Oscillospira* (STD-D: 5.03%, CAF-D: 1.93%; *q* = 0.027), and *Dehalobacterium* (STD-D: 0.16%, CAF-D: 0.05%; *q* = 0.047), and an uncharacterized genus of the *Rikenellaceae* family in the *Bacteroidales* order (STD-D: 2.17%, CAF-D: 0.83%; *q* = 0.019).

**Figure 2:**
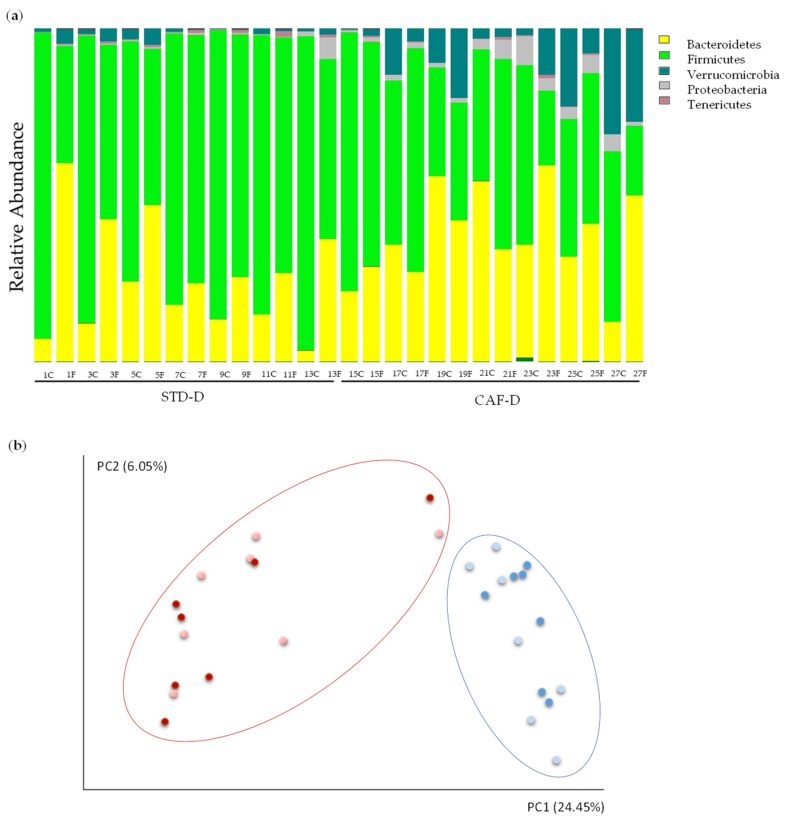
Changes in the metagenome of rats fed with cafeteria diet (CAF-D) and chow diet (STD-D): (**a**) the 5 phyla represented by relative abundance and (**b**) analysis of beta diversity represented by scores of each STD-D (blue) and CAF-D (red) groups in FCS (light color) and CEC (dark color) after Principal Coordinate Analysis (PCoA) with unweighted UniFrac.

Alpha diversity values, i.e., measures of variability within a sample, were calculated with a variety of indices that measure richness and variation (including Shannon, Simpson, chao1, observed OTUs index, and phylogenetic diversity) (Figure S1). Shannon and Simpson indices showed evenness in the population of both groups. The observed OTUs index was significantly decreased in FCS in the CAF-D group, though it was non-significantly decreased in CEC (FCS *p* = 0.036; CEC *p* = 0.066). However, chao1 was significantly lower in both, FCS and CEC (FCS *p* = 0.036; CEC *p* = 0.048). In addition, the phylogenetic diversity was significantly lower in CAF-D (FCS *p* = 0.030; CEC *p* = 0.012). The estimation of beta diversity, i.e., an indication of variability among groups, by means of a Principal Coordinate Analysis (PCoA) (Figure 2b) showed a clear and statistically significant separation between the STD-D and CAF-D groups (*q* < 0.001).

### 3.2. Depletion of Microbiota in Receptor Animals after the Antibiotic Treatment

After the obtention of the donors cecal content, the transplant in healthy animals was performed. Previously, the experimental procedure requires a previous depletion of the host microbiota by means of an antibiotic treatment, and a subsequent metagenomic analysis of the FCS to evaluate the success of the depletion. The reads count in 16S rRNA gene sequencing were 188–342.242 per sample. The low minimum reads corresponds with a low quantity of bacterial DNA, which was also difficult to amplify. Data from the STD-D group were used to compare the microbiota after the antibiotic treatment. At the phylum level, all FCS samples had similar taxonomic relative abundance, composed mainly by *Firmicutes* (57%) and *Bacteroidetes* (30.09%) (Figure S2a). Other less abundant phyla included *Proteobacteria* (3.8%), *Verrumicomicrobia* (2.6%), *Spirochaetes* (1.6%), *Actinobacteria* (1%), *Cyanobacteria* (1%), and additional phyla not listed due to represent <1%. Thus, there is an emergence of less abundant phyla and a decrease in most abundant phyla (Figure S2b). Alpha diversity indices confirmed the decrease in bacteria after the treatment (Figure S2c). More concisely: (1) FCS samples showed lower levels of OTUs index per genus compared to the STD-D group; (2) Shannon and Simpson indices were 6.63 ± 0.12 and 0.99 ± 0.002, respectively; (3) the average of observed OTUs (124 ± 5.90) and chao 1 (465.65 ± 55.86) in the FCS were three orders of magnitude lower compared to the STD-D group; and 4) the phylogenetic diversity was 14.69 ± 1.38, being values too low compared to other studies. Moreover, the PCoA confirmed the homogeneity of the beta diversity of the host microbiota after the depletion treatment with antibiotics (Figure S2d).

### 3.3. Metagenomic Characterization of the Model of Obesity-Associated Gut Dysbiosis with a Healthy Phenotype

The biometric and biochemical parameters, plasma parameters, and liver biochemistry, which are summarized in Table S2, were carried out to better characterize the model of obesity-associated gut dysbiosis in the context of a healthy phenotype. Focusing on biometric parameters, the total white adipose tissue weight (gr.) increased in CAF-R group compared to the STD-R group; concretely a tendency to increase in MWAT (STD-R: 4.04 ± 0.26, CAF-R: 5.16 ± 0.52; *p* = 0.09) was observed and a slight increase in RWAT (STD-R: 7.14 ± 0.90, CAF-R: 9.70 ± 1.70; *p* = 0.22) was also assessed. Once the transplant was done, the effect of the cecal content transplant was mainly observed by a significant decrease in CEC weight (gr.) in the cecal content receptors groups versus the control group (CNT-R: 8.43 ± 0.63, STD-R + CAF-R: 5.28 ± 0.32; *p* = 0.002). Some plasma parameters presented a tendency to increase, including TG (STD-R: 80.91 ± 14.01, CAF-R: 117.30 ± 17.12; *p* = 0.1), TC (STD-R: 44.81 ± 9, CAF-R: 68.19 ± 11; *p* = 0.1), and NEFAs (STD-R: 0.37 ± 0.02, CAF-R: 0.43 ± 0.03; *p* = 0.1), while glucose remained unaltered. However, total liver lipids (STD-R: 38.57 ± 2.20, CAF-R: 28.99 ± 2.22; *p* = 0.01) significantly decreased in the CAF-R group, and specifically phospholipids (STD-R: 12.85 ± 0.60, CAF-R: 10.67 ± 0.67; *p* = 0.03) presented the highest decrease.

**Figure 3:**
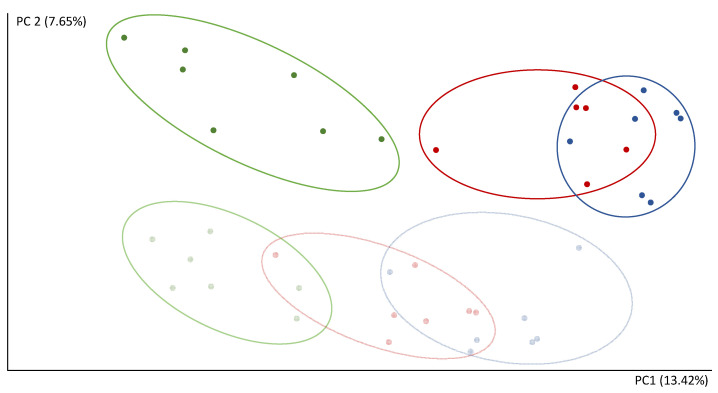
Analysis of beta diversity after the transplant represented by scores after PCoA (unweighted UniFrac). Green, CEC CNT-R; light green, FCS CNT-R; blue, CEC STD-R; light blue, FCS STD-R; red, CEC CAF-R; light red, FCS CAF-R.

The reads count in 16S rRNA gene sequencing were 151.796–522.785 per sample. After the microbiota transplant, the composition of the communities showed a separation of the FCS and CEC samples in the second component (PC2) of the PCoA (Figure 3). The first component (PC1) of the PCoA clearly separates CNT-R and the transplanted rats (STD-R and CAF-D) (Figure 3). Thus, PC1 and PC2 explain 13.42% and 7.65% of the variability, respectively. Furthermore, the CNT-R group versus the transplanted microbiota rats (STD-R and CAF-D) had a smaller number of species, as shown by a significant decrease in the alpha diversity indices in CEC, i.e., chao1, observed OTUs and phylogenetic diversity. This decrease was not-significantly decreased in FCS (Figure S3). 

**Figure 4:**
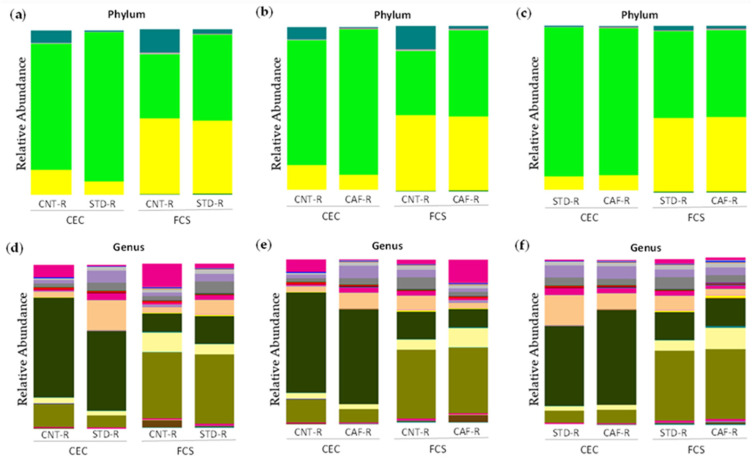
Microbiota composition between CNT-R, STD-R, and CAF-R groups represented by abundance: (**a**) phylum level in CNT-R vs. STD-R, (**b**) phylum level in CNT-R vs. CAF-R, (**c**) phylum level in STD-R vs. CAF-R, (**d**) genus level in CNT-R vs. STD-R, (**e**) genus level in CNT-R vs. CAF-R, (**f**) genus level in STD-R vs. CAF-R.

At phylum level, the effect of the transplant in STD-R promoted an establishment of *Firmicutes* in CEC (CNT-R: 71.82% vs. STD-R: 91.02%; *p* = 0.05; *q* = 0.09) and in FCS (CNT-R: 40.5% vs. STD-R: 52.59%; *p* = 0.14; *q* = 0.28), whereas *Bacteroidetes* remained low in CEC (CNT-R: 18.86% vs. STD-R: 7.86%; *p* = 0.15; *q* = 0.18) and almost unaltered in FCS (CNT-R: 44.88% vs. STD-R: 43.2%; *p* = 0.79; *q* = 0.79). Otherwise, minor represented phyla such as *Verrucomicrobia* (CNT-R: 8.46% vs. STD-R: 0.78%; *p* = 0.02; *q* = 0.07), *Proteobacteria* (CNT-R: 0.66% vs. STD-R: 0.21%; *p* = 0.05; *q* = 0.09), and *Actinobacteria* (CNT-R: 0.14% vs. STD-R: 0.03%; *p* = 0.008; *q* = 0.05) were decreased in CEC. The sorted minor phyla had more presence in CNT-R CEC instead of STD-R CEC compensating the non-establishment of the two major phyla (Figure 4a). Focusing on genera, the differences between CNT-R and STD-R are summarized in Table S3. Thus, the differences in *Firmicutes* were characterized in the cecum of STD-R group by the significative alteration of *Ruminococcus*, *Oscillospira*, and *Coprococcus* and an uncharacterized genus in the *Clostridiales* order (Figure 4d). Moreover, some differences were also found in feces of STD-R group characterized by changes in rc4-4 genus and an uncharacterized genus. 

Regarding the CAF-R group, the differences were similar as previously described for the STD-R group with the establishment of *Firmicutes* in CEC (CNT-R: 71.82% and CAF-R: 89.3%) and in FCS (CNT-R: 40.5% and CAF-R: 52.05%), while *Bacteroidetes* remain low in CEC (CNT-R: 18.86% and CAF-R: 9.14%) and almost equal in FCS (CNT-R: 44.88% and STD-R: 43.89%). Besides, *Verrucomicrobia* phylum was increased in the CNT-R group having more differences in CEC than in FCS group (Figure 4b). Focusing on genera, the differences between CNT-R and CAF-R are summarized in Table S4. Thus, *Oscillospira* genus was the main altered genus presenting a significative increase in both sample types among other interesting changes in CEC genera as *Coprococcus* and *Ruminococcus* (Figure 4e).

The receptors of cecal content, the STD-R and the CAF-R groups, had a similar phyla composition (Figure 4c). Focusing on genera, the statistical analysis between STD-R and CAF-R are summarized in Table S5. Although the animals presented a similar phyla composition, some differences could be observed in genera as it is shown in Figure 4f. In this case, more differences were observed in FCS than in CEC. In the case of CEC, an uncharacterized genus of *Lachnospiraceae* family presented a significant one-half decrease in CAF-R. Although there were not any more statistically significant differences, there were some genera with interesting fold changes as the case of *Parabacteroides*.

### 3.4. Metabolomic Characterization of the Model of Obesity-Associated Gut Dysbiosis with a Healthy Phenotype

The plasma metabolomic approach was based on a multiplatform global analysis including 139 metabolites belonging to: the metabolism of lipids as a wide diversity of different triglycerides (TG), ester cholesterols (ChoE), diacylglycerols (DG), sphingomyelins (SM), phosphatidylcholines (PC), and lysophospholipids (LPC); metabolism of carbohydrates as the main metabolites of citric acid pathway were included; and metabolism of the main amino acids affecting the microbiota and diet were included among other interesting metabolites. The analysis showed differences on the lipid metabolism in the CAF-R group in comparison to the STD-R group (Table S6). After the parametric unpaired *t*-test, 7 different significant lipids were determined as potential biomarkers in plasma (DG 34:2, DG 34:3, DG 36:2, DG 36:4, LPC 20:0, DG 34:1, and PC 31:0). However, only one plasma metabolite was significantly differentiated after the multivariate correction, which was the DG 34:2. Specifically, the DG 34:2 was significantly increased in CAF-R compared to CNT-R (*q* = 0.009) and STD-R (*q* = 0.045) (Table 1). Moreover, DG 34:2 is the most important feature in the model after applying the Random Forest classifier presenting the highest value by far in comparison to the second metabolite in the list (Table S7).

The urine metabolomic approach was based on untarget ^1^H-NMR methodology detecting 45 metabolites belonging, mainly, to the metabolism of amino acids (e.g., phenylalanine, tyrosine, and tryptophan metabolism; glycine, serine, and threonine metabolism; alanine, aspartate, and glutamate metabolism; glutathione metabolism; and taurine and hypotaurine metabolism) and the energetic metabolism (e.g., citrate cycle, pyruvate metabolism, and glycolysis/gluconeogenesis) (Table S8). After the parametric unpaired *t*-test, 6 different significant metabolites were determined as potential biomarkers in urine (hippurate, *o*-coumaric acid, 3-HPPA, HPPA sulfate, tyrosine, and phenylacetylglycine). After the multivariate correction, the results pointed out three metabolites involved in the phenylalanine metabolism that were significantly altered in the CAF-R group compared to the STD-R group (hippurate, *o*-coumaric acid, 3-HPPA). On the one hand, the *o*-coumaric acid (*q* = 0.035) and the 3-hydroxyphenylpropionate (3-HPPA) (*q* = 0.039) were significantly increased in the CAF-R group compared with the STD-R group, almost 3 and 10 times more elevated, respectively. On the other hand, the hippurate (*q* = 0.013) was significantly decreased by a half in the CAF-R group in comparison to the STD-R group (Table 1). Moreover, those metabolites are the most important features in the model after applying the Random Forest classifier being the top metabolites to discern between the STD-R and CAF-R groups (Table S9).

### 3.5. Correlation between Metagenomics and Metabolomics in the Obesity-Associated Gut Dysbiosis

**Figure 5:**
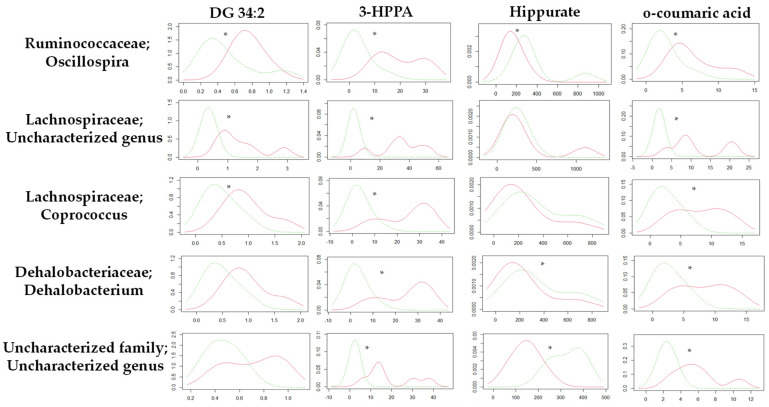
Kernel density plot of the altered metabolites normalized by genus for the STD-R and CAF-R group. The statistical comparisons between metabolites normalized by genus were conducted using test of equal densities. The X axis represents the values of Kernel density while the Y axis represents the metabolite values. Each row represents the metabolites normalized by one selected genus. Each column shows the metabolites represented in the Kernel density plot. The significant differences are highlighted by an asterisk. Green line: STD-R; Red line: CAF-R.

Focusing on the metabolic differences between the STD-R and the CAF-R groups, none of the metabolites (n = 4) used in this study were correlated with values of metagenomic diversity (Table S10). Nevertheless, we focused on specific genus. In this case, the Kernel density distribution of altered metabolites was correlated with some genus normalizing the relative values of the metabolites by the different genus, discerning between STD-R and CAF-R groups. Thus, 28 genera with a higher abundance of 0.1% were selected to study the density distribution. Interestingly, the density distribution of the STD-R and the CAF-R groups was significantly different in 3 genera for DG 34:2, 19 genera for 3-HPPA, 9 genera for Hippurate, and 14 genera for *o*-coumaric acid (Table S11). Indeed, *Firmicutes* was the phylum with the great part of genus affecting the distribution between groups. For example, the *Oscillospira* genus was differently distributed between groups in the four selected metabolites (Figure 5), and differences were found in donors and receptors. In addition, besides *Oscillospira* genus, other genera from the *Clostridiales* order have at least 3 altered metabolites, i.e., *Coprococcus* and an uncharacterized genus of *Lachnospiraceae* family; *Dehalobacterium* genus of *Dehalobacteriaceae* family; and an uncharacterized genus of uncharacterized family (Figure 5). 

## 4. Discussion

In the present study, a pilot metabolomic approach in healthy rats, that received cecal microbiota from obese ones, has been carried out to find a metabolic profile of obesity-associated GM with a healthy phenotype, avoiding metabolic disturbances related to other risk factors. Importantly, the transference of GM from obese to non-obese rats could help to discover new biomarkers exclusively related to this GM alterations that could provide interesting information about the metabolic profile of a segregated obesity-associated gut dysbiotic state.

Interestingly, focusing on biometric parameters, a huge significant decrease in CEC weight was observed which could be directly induced by the effect of the cecal content. Additionally, there was a tendency to increase in the total weight of white adipose tissue, which was more evident in MWAT although a slight increase was also observed in RWAT. Thus, the transplanted cecal content affected the weight of the white adipose tissue. Focusing on plasma biochemistry, the glucose levels remained unaltered but TG, TC, and NEFAs presented a clear tendency to increase in the CAF-R group supporting the changes observed in MWAT and RWAT. On the other hand, the total liver lipids decreased in the CAF-R group, and specifically the phospholipids were the representative lipid species that also correlated with this decrease. Globally, all these changes demonstrate the impact of the different cecal donors’ content in transplant on some biometric and biochemical parameters. 

In our study, the depletion of host microbiota after the antibiotic treatment, which produces a decrease in the number of microbes, was characterized by an emergence of less abundant phyla and a decrease in most abundant phyla in receptors animals (CNT-R, STD-R, and CAF-R). In our pilot model of obesity-associated gut dysbiosis in rats, we altered the microbiota of cecal receptors (STD-R and CAF-R) by oral gavage of cecal donors content (STD-D and CAF-D), which were fed with a standard diet. First, to determine the success of the transplant, the control group (CNT-R) and CEC receptors groups were compared (STD-R and CAF-R). Results showed an increase of bacteria and diversity in CEC receptors groups, as well as a decrease in CEC weight. Moreover, we observed slight differences between STD-R and CAF-R, where there was a change in the distribution of bacteria. Significant increases in genera of the STD-R group were induced in the *Clostridiales* spp.; *Ruminococcus*, *Oscillospira*, *Coprococcus*; and an uncharacterized genus (which were increased in CEC). On the other hand, an uncharacterized genus of *Ruminococcaceae* and rc4-4 genus was significantly increased in FCS. In the case of CAF-R versus CNT-R, *Oscillospira* genus was increased either in CEC or FCS following the trend of STD-R. The differences in *Oscillospira* genus were maintained between the donors and receptors, which has been defined as a component of the GM related to leanness or lower BMI, confirming our model of obesity-associated to GM in a healthy phenotype [47]. Finally, some minor changes in the genera could be observed, including some important genera in the development of obesity (e.g., changes in the composition of *Clostridiales* spp.). Although, clear statistical differences between STD-R and CAF-R groups were not observed in metagenomics analysis, if we consider all these changes together with the biometric and biochemical parameters, we could sense a segregated model of obesity-associated gut dysbiosis.

In addition, some metabolic changes were observed in the host, induced by the alteration of the complexity microbial biofilm. In fact, the most interesting altered metabolites included those in plasma (e.g., DG 34:2) and urine (e.g., Hippurate, 3-HPPA, and *o*-coumaric acid), pointing out urine as a fundamental part of the metabolic profile of our model. These metabolic variations provide another hint to prove the achievement of the segregated dysbiosis between STD-R and CAF-R groups. In this sense, taking together the biometric and biochemical parameters, the metagenomics and, finally, the metabolomics, the general picture of the model would be elucidated. Thus, we can consider the experiment as a successful pilot model of obesity-associated gut dysbiosis. 

DG were the main metabolites with altered circulating plasma levels that were found in transplanted rats; being increased in CAF-R compared to CNT-R and STD-R. However, after the multivariate correction, only the DG 34:2 was statistically significant. DG are glycerides consisting of two fatty acid chains covalently bonded to a glycerol molecule through ester linkages [48] and, apart from being the central intermediate in the synthesis of membrane phospholipids and the lipid storage [49], they are key regulators of cell physiology, controlling the membrane recruitment and activation of signaling molecules [50]. For example, Backhed and collaborators have suggested that because of dysbiosis, GM can stimulate the levels of TG and DG through the suppression of the intestinal epithelial expression of the fasting-induced adipose factor (Fiaf), a natural inhibitor of circulating lipoprotein lipase (LPL) [51], which is the main rate-limiting enzyme in lipid metabolism, catalyzing the hydrolysis of TGs and DG [52]. Our results suggest that microbial alteration associated to obesity could stimulate Fiaf expression, potentiating the inhibition of LPL and therefore, increasing the circulating levels of DG, specifically the DG 34:2. In this sense, the DG 34:2 is a diacylglycerol with fatty acids containing a total of 34 carbons and 2 double bonds joined via ester linkages at unknown positions (sn1, sn2, or sn3), it is mainly implicated in the novo triacylglycerol biosynthesis of several TG as other DG [53]. This specific lipid has been attracting attention in studies of lipidomics in diverse fields focusing on, e.g., diabetic kidney tissue of diabetic rats [54] and liver tissue of hypertensive rats [55]. Despite this metabolite was found as a biomarker in several pathologies and tissues in rats as it has been described before, we also propose this specific lipid to do further studies in obesity-associated to gut dysbiosis. Although the first lipidomic biomarkers are entering in the clinic, certain analytical standards need to be established in order to make lipidomic measurements generally accepted in clinical settings [56]. However, tissues responsible for DG levels in plasma are still unknown. Several authors have pointed out the utility of increased levels of plasma DG, as well as its composition, as biomarkers of metabolic syndrome and obesity in rodents [57,58], rhesus monkeys [59], and humans [60], although without specifying the type/s of DG.

On the other hand, a relevant finding in our pilot study is the alteration on hepatic lipids observed in transplanted animals. CAF-R group showed decreased levels of total hepatic lipids and phospholipids compared to STD-R and CNT-R groups. Interestingly, it has been recently shown [61] that the transfer of dysbiotic gut microbiota from obese to antibiotic-free conventional mice changes gut microbiota and microbiome of recipient mice, ameliorates hepatic gluconeogenesis, and prevents high-fat diet-induced dysmetabolism. These results are in agreement with our present results and point out the potential success of our dysbiosis model. Additionally, despite not directly related to microbiota transfer, another interesting study [62], where the connection of the antifungal carbendazim (CBZ) on lipid metabolism was studied discovered that CBZ chronic treatment induced gut microbiota dysbiosis in mice and such dysbiosis was associated with a reduced lipid liver synthesis and an increased lipid storage in the fat. More concisely, regarding the liver, some genes involved in TG synthesis, such as Dgat1 and Gpat, were significantly downregulated by the CBZ chronic treatment. 

Furthermore, there are, mainly, two enzymes, the diacylglycerol acyltransferase (DGAT) and ethanolamine phosphotransferase, which control the use of DG for lipid synthesis, suggesting the presence of a common DG pool for lipid synthetic pathways [63]. Another DG pool is also available for glycerolipid synthesis because DG that is released from TG stores in human fibroblasts can be converted to phospholipids [64]. Segregation of DG toward different metabolic routes seems to occur according to the cell’s needs. For instance, the DG originally destined to form phospholipids is re-directed toward TG when phospholipid synthesis is inhibited [65]. In our animal model, the obesity-associated gut dysbiosis model produces a significant increase in plasma DG levels, as the DG 34:2, and a decrease in hepatic phospholipids. Thus, it would be expected an increase in plasma or liver TG, although they were not changed. Further studies are needed to elucidate the relation between microbes, DG 34:2, and liver phospholipids.

The obesity-associated to gut dysbiosis in a healthy phenotype also produced the alteration of three main metabolites, i.e., hippurate, *o*-coumaric acid, and 3-HPPA, in urine. These metabolites, which belong to the phenylalanine metabolism, have also been related to the degradation of phenolic compounds that have been traditionally associated with the ingestion of polyphenols-rich food [66]. In this sense, diet is one of the major environmental factor that modulates the composition and the metabolic activity of GM, forming the food–gut axis [67]. Polyphenols are plant secondary metabolites, and there are many studies that support the idea that phenolic compounds modulate the composition and metabolic activities of GM, as well as GM metabolize polyphenols into bioactive compounds that produce clinical benefits [68,69]. Hence, it has been postulated that changes in the species population or GM activities result in changes in the metabolic processing of polyphenolic compounds that can be observed in the derived urinary metabolites [70]. 

In our case, the changes in urine metabolomics are explained by the microbiota transplant and not by the modulation of dietary polyphenols, because all the animals received the same diet (without differences in trace polyphenols). The phenolic compounds that have been found altered in our urine model are included in the group of chlorogenic acids, standing out the contradictory information in literature about the bioavailability and effects of these type of polyphenols [66]. Interestingly, Clayton and collaborators proposed two distinct rat urinary compositional phenotypes, i.e., these may arise from differences in the gut microbially mediated metabolism of phenylalanine that are characterized by differences in hippurate and 3-HPPA, among other metabolites [71]. 

Hippurate is a glycine conjugate of benzoic acid formed in the mitochondria of the liver and kidneys and then excreted in the urine [72] and is considered a gut microbial-mammalian co-metabolite that can be made by *Clostridium* spp., primarily from polyphenols [73]. In our study, hippurate was reduced by a half in the CAF-R group compared to the STD-R group. In this sense, hippurate excreted in urine has been found as a distinguishing feature of different range on physiological and pathological conditions (e.g., obese phenotypes [74,75], metabolic syndrome [76], Crohn’s disease [77], psychological disorders [78], among others). Additionally, many studies have shown an increased excretion of hippurate resulted from the ingestion of specific dietary components containing phenolic molecules, as teas [79,80] or edible fruits [81]. Taking into account the previous information, hippurate could be considered as a biomarker of health but this concept is ambiguous, since the source has not been robustly addressed. Thus, the wide associations of hippurate to different conditions support the idea of our findings that the changes in its excretion are caused by the GM and not by the disease. These results are in agreement with a recent review showing that the differences in hippurate excretion are due, at least in part, to functional or compositional differences in GM, regardless of the specific diet [70].

Related to hippurate, the 3-HPPA is a phenol derivative formed through fermentation of tyrosine by *Clostridium* spp., that could be further metabolized to benzoic acid and excreted as hippurate [82,83]. Interestingly, related to chlorogenic acids availability, 3-HPPA has been shown to be able to freely cross the gut epithelium [84] into the blood and brain [85]. In our animal model, 3-HPPA was increased almost 10 times in the CAF-R group compared to the STD-R. In a recent study, the effect of procyanidin A2, and its major colonic metabolite 3-HPPA, was investigated on the suppression of macrophage foam cell formation. The results showed a significant reduction in the cellular lipid accumulation and the inhibition of foam cell formation by both compounds [86]. According to our study, we speculate that the increased level of 3-HPPA urine excretion in CAF-R could be related to the reduction in lipid accumulation because CAF-R showed a lower 3-HPPA compared to STD-R. However, this mechanism would not directly explain the reduction in total lipids in liver showed in CAF-R animals. Taking all of these data into consideration, we hypothesize that 3-HPPA could be a direct explanation of the decrease in hippurate because of elevated excretion levels in the urine profile. In agreement, there was no 3-HPPA availability in the CAF-R animals to metabolize hippurate. Furthermore, dietary modulation was found to cause a change in the excretion of 3-HPPA, which was replaced by hippurate in Wistar rats [87]. Surprisingly, the excretion of hippurate persisted when the animals returned to the original diet. It was proposed that, in addition to the precursors available in the diet, the absence or presence of urinary hippurate and 3-HPPA was influenced by variation on the GM. Additionally, this research proposed that a change in diet could potentially have caused a redistribution of the microbiota, resulting in the production of hippurate as the primary excretion product, regardless of the specific diet [87].

*o*-coumaric acid, which is an hydroxycinnamic acid, has been described to act as powerful antioxidant and as an important biological protector from oxidation [88]. Interestingly, our findings showed that *o*-coumaric acid excretion in urine was increased three times in the CAF-R group compared to the STD-R. Related to this, a research performed in rats fed with high-fat diet (HFD) showed that supplementing the HFD with *o*-coumaric acid for 8 weeks suppressed the increases in body weight, liver weight, and adipose tissue weights of peritoneal and epididymal fat induced by the hypercaloric diet [89]. Thus, in our case, the high increase in the *o*-coumaric secretion in urine may be hypothetically related to a decreased systemic protective effect in the CAF-R group, having, in consequence, a predisposition to develop obesity in the future. As far as we know, this is the first time that *o*-coumaric acid is proposed as a dysbiosis biomarker.

Finally, some correlations will be discussed to directly connect the obesity-associated gut dysbiosis with the host metabolism. There was a significant correlation between some genera in *Clostridiales* order and the metabolites included in the profile of metabolic changes. The *Oscillospira* genus was highly correlated with the selected metabolites followed by other genus with three out of four metabolites including the following genera: *Coprococcus* and an uncharacterized genus of *Lachnospiraceae* family, *Dehalobacterium* genus of *Dehalobacteriaceae* family, and an uncharacterized genus of uncharacterized family. Previous intervention studies in humans showed minor effect on the metabolism of phospholipids and cholesterol (in large VLDL), after changes in the metagenomic composition induced by moderate exercise [90]. Other studies have shown correlation of metagenomics with imbalanced metabolome, resulting in a source of potential biomarkers of obesity [91], chronic obstructive disease in humans [92], the dietary effect of the insulin feeding in pigs [93], the quantifying diet effect in humans [94], or tracking a healthy dietary pattern [95]. Thus, external factors that induce changes in the microbial community, lead to changes in the metabolism. 

Previous studies have found that changes in the microbiota produce metabolic alterations, however, to the best of our knowledge, this is the first study that focuses on changes in the metabolism caused by obesity-associated gut dysbiosis with a healthy phenotype. This study, under controlled experimental conditions, elucidates the metabolic changes caused by an obesity-associated gut dysbiosis. This fact opened a window of opportunities to propose metabolic biomarkers of segregated obesity-associated gut dysbiosis in a healthy population.

## 5. Conclusions

The important point in the present study is that we have developed a pilot experiment trying to isolate dysbiosis from the rest of obesity-associated complications (e.g., hyperglycemia, hyperinsulinemia, hyperlipidemia, hypercoagulable state, etc.). In this sense, we have been able to discriminate the alterations induced by the dysbiosis component of obesity in a relative isolated way. Our model of obesity-associated microbial gut dysbiosis in healthy rats produced biometric and biochemical changes, as well as metabolic changes, mainly in the lipid (DG 34:2 in plasma) and phenylalanine (hippurate, 3-HPPA, and *o*-coumaric acid in urine) metabolism. In consequence, we propose that external factors that induce changes in the microbial community may trigger the mechanism of obesity by altering mainly the lipid and phenylalanine metabolism of the host. To the best of our knowledge, this is the first study proposing this model of a segregated risk factor of obesity, expanding, in consequence, the knowledge about the metabolism on obesity-associated microbial gut dysbiosis as well as the determination of a metabolic profile of the risk factor. Hereby, we propose an obesity-associated metabolic profile, including DG 34:2, hippurate, 3-HPPA, and *o*-coumaric, that can be utilized as tentative biomarkers of an obesity-prone state mainly related to a dysbiotic state. These pilot approach and associated results provide the basis for a better understanding of the biological role played by GM and for the discovery of novel biomarkers in future obesity studies.

## Figures and Tables

**Table 1:** Metabolites significantly altered affected by the microbiota transplant in plasma and urine. The statistically significant *p*-values (*p* < 0.05) and q-values (*q* < 0.05) are highlighted in bold. DG 34:2, diacylglycerol 34:2; 3-HPPA, 3-hydroxyphenylpropionate.

	Biofluid	Plasma	Urine	Urine	Urine
	Metabolite	DG 34:2	Hippurate	*o*-Coumaric Acid	3-HPPA
**Mean ± S.E.M.**	CNT-R	0.40 ± 0.03	192.13 ± 48.87	4.36 ± 0.84	15.58 ± 5.87
	STD-R	0.42 ± 0.04	295.91 ± 20.55	2.16 ± 0.20	2.31 ± 0.60
	CAF-R	0.72 ± 0.05	145.49 ± 21.45	6.14 ± 0.76	21.30 ± 3.91
**CNT-R vs. STD-R**	*p*-value	0.549	0.086	**0.039**	0.064
	*q*-value	0.963	0.351	0.290	0.290
	FC	1.07	1.54	0.50	0.15
**CNT-R vs. CAF-R**	*p*-value	**<0.001**	0.407	0.141	0.435
	*q*-value	**0.009**	0.770	0.770	0.783
	FC	1.82	0.77	1.41	1.37
**STD-R vs. CAF-R**	*p*-value	**<0.001**	**<0.001**	**0.002**	**0.003**
	*q*-value	**0.045**	**0.013**	**0.035**	**0.039**
	FC	1.69	0.49	2.84	9.24

Bold figures mean significant.

## Data Availability

The data presented in this study are available on request from the corresponding author.

## Supplementary Materials

The following are available online at https://www.mdpi.com/2218-273X/11/2/303/s1, Figure S1: Characterization of the alpha diversity indices derived from the QIIME command α rarefraction of rats fed with a standard (STD) and cafeteria (CAF) diets in different metagenomic biofluids (cecum (CEC) and feces (FCS)); Figure S2: Microbiota analysis after the antibiotic treatment; Figure S3: Characterization of the alpha diversity indices derived from the QIIME command α rarefraction after the microbiota transplant in different metagenomic biofluids (CEC and FCS); Table S1: Summary of metagenomics in the standard diet donors (STD-D) and the cafeteria diet donors (CAF-D) groups in CEC and FCS focusing on taxonomic data; Table S2: Biometric parameters, plasma parameters and liver biochemistry of transplant model; Table S3: Summary of metagenomics in the control group (CNT-R) and the standard receptors (STD-R) groups in CEC and FCS focusing on taxonomic data; Table S4: Summary of metagenomics in the CNT-R and the cafeteria receptors (CAF-R) groups in CEC and FCS focusing on taxonomic data; Table S5: Summary of metagenomics in the STD-R and the CAF-R groups in CEC and FCS focusing on taxonomic data; Table S6: Statistical analysis of plasma metabolites in the STD-R and the CAF-R groups; Table S7: Plasma feature importance of Random Forest Classifier; Table S8: Statistical analysis of urine metabolites in the STD-R and the CAF-R groups; Table S9: Urine feature importance of Random Forest Classifier; Table S10: Correlation between altered metabolites and alpha diversity; Table S11: Summary table of the relation between altered metabolites and genus.
