# Supplementary material for: A Pilot Study for Metabolic Profiling of Obesity-Associated Microbial Gut Dysbiosis in Male Wistar Rats
Source: Biomolecules. 2021 Feb 18;11(2):303. doi: 10.3390/biom11020303 (PMC7922951; doi:10.3390/biom11020303)
Supplement: Supplementary file 1 [file biomolecules-11-00303-s001.pdf]

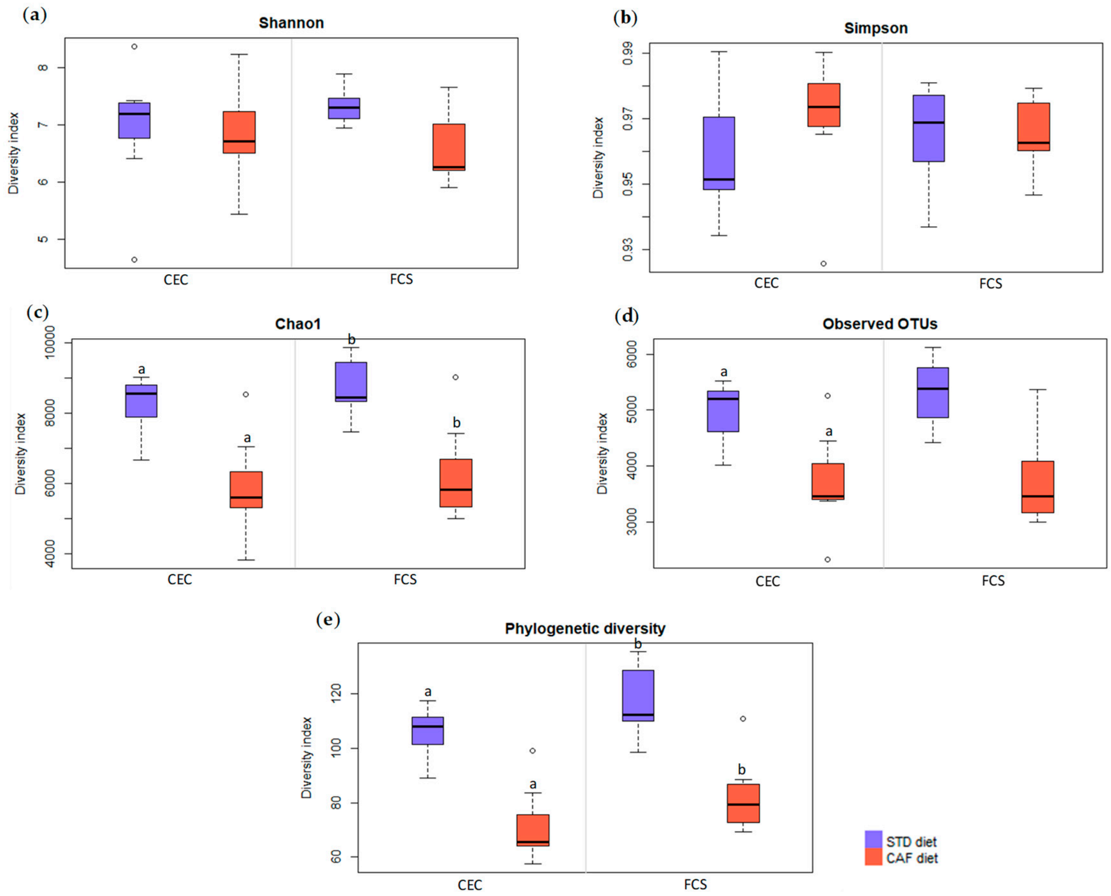

Figure S1. Characterization of the alpha diversity indices derived from the QIIME command α rarefraction of rats fed with a STD (blue boxplots) and CAF diet (red boxplots) in different metagenomic biofluids (CEC and FCS): (**a**) Shannon index, (**b**) Simpson index, (**c**) Chao1, (**d**) Observed OTUs, (**e**) Phylogenetic diversity. Different lowercase letters a and b indicate significant ($p < 0.05$) changes between diets.

Table S1. Summary of metagenomics in the STD-D and the CAF-D groups in CEC and FCS focusing on taxonomic data. Taxonomic data presented as the mean ± S.E.M. (n=7) per group shorted by q-value of CEC. The summary of univariant analysis is shown including p-value, q-value and FC, the statistically significant p-values and q-values (< 0.05) are highlighted in bold.

					**CEC**					**FCS**				
**Phylum**	**Class**	**Order**	**Family**	**Genus**	**STD-D (%)**	**CAF-D (%)**	***p*-value**	***q*-value**	**FC**	**STD-D (%)**	**CAF-D (%)**	***p*-value**	***q*-value**	**FC**
Firmicutes	*Clostridia*	*Clostridiales*	-	-	53.47 ± 5.66	9.31 ± 3.54	**<0.01**	**<0.01**	0.17	29.73 ± 4.17	5.59 ± 1.84	**<0.01**	**0.02**	0.19
Firmicutes	*Clostridia*	*Clostridiales*	*Lachnospiraceae*	*Ruminococcus*	0.69 ± 0.19	1.96 ± 0.2	**<0.01**	**0.01**	2.83	0.55 ± 0.1	1.05 ± 0.18	**0.03**	0.13	1.93
Firmicutes	*Bacilli*	*Turicibacterales*	*Turicibacteraceae*	*Turicibacter*	0.14 ± 0.03	0.02 ± 0.01	**<0.01**	**0.02**	0.13	0.15 ± 0.06	0.26 ± 0.12	0.42	0.58	1.76
Bacteroidetes	*Bacteroidia*	*Bacteroidales*	*Porphyromonadaceae*	*Parabacteroides*	0.22 ± 0.08	3.92 ± 0.81	**<0.01**	**0.03**	17.86	0.42 ± 0.1	3.44 ± 0.75	**0.01**	0.05	8.15
Firmicutes	*Clostridia*	*Clostridiales*	*Lachnospiraceae*	*Blautia*	0.12 ± 0.02	2.63 ± 0.62	**0.01**	**0.04**	21.65	0.08 ± 0.02	2.59 ± 0.81	**0.02**	0.1	32.14
Bacteroidetes	*Bacteroidia*	*Bacteroidales*	*S24-7*	-	9.61 ± 2.04	23.43 ± 4.04	**0.01**	0.07	2.44	33.08 ± 4.8	33.9 ± 3.51	0.89	0.93	1.02
Firmicutes	*Clostridia*	*Clostridiales*	*Peptostreptococcaceae*	-	0.58 ± 0.15	0.12 ± 0.05	**0.02**	0.09	0.2	0.53 ± 0.34	0.63 ± 0.2	0.82	0.92	1.17
Bacteroidetes	*Bacteroidia*	*Bacteroidales*	*Bacteroidaceae*	*Bacteroides*	0.65 ± 0.14	5.98 ± 1.81	**0.03**	0.09	9.13	1.62 ± 0.33	1.96 ± 0.27	0.44	0.58	1.21
Firmicutes	*Clostridia*	*Clostridiales*	*Peptococcaceae*	*rc4-4*	0.39 ± 0.1	0.96 ± 0.2	**0.03**	0.11	2.48	0.25 ± 0.04	0.22 ± 0.06	0.65	0.79	0.86
Proteobacteria	*Gammaproteobacteria*	*Enterobacteriales*	*Enterobacteriaceae*	-	0.29 ± 0.18	3.06 ± 1.08	**0.04**	0.12	10.68	1.13 ± 0.86	2.72 ± 0.78	0.2	0.42	2.4
Verrucomicrobia	*Verrucomicrobiae*	*Verrucomicrobiales*	*Verrucomicrobiaceae*	*Akkermansia*	1.21 ± 0.36	12.01 ± 4.5	**0.05**	0.12	9.94	2.28 ± 0.75	11.11 ± 3.82	0.06	0.21	4.88
Firmicutes	*Erysipelotrichi*	*Erysipelotrichales*	*Erysipelotrichaceae*	*Eubacterium*	0	0.11 ± 0.04	**0.05**	0.12	76.02	0	0.17 ± 0.09	0.1	0.27	39.76
Tenericutes	*Mollicutes*	*RF39*	-	-	0.25 ± 0.05	0.09 ± 0.05	0.06	0.13	0.38	0.6 ± 0.18	0.43 ± 0.15	0.48	0.61	0.72
Firmicutes	*Clostridia*	*Clostridiales*	*Ruminococcaceae*	*Oscillospira*	5.62 ± 0.84	3.55 ± 0.72	0.09	0.17	0.63	5.03 ± 0.63	1.93 ± 0.54	**<0.01**	**0.02**	0.38
Firmicutes	*Clostridia*	*Clostridiales*	*Lachnospiraceae*	*Dorea*	0.1 ± 0.01	0.24 ± 0.07	0.11	0.2	2.42	0.13 ± 0.03	0.28 ± 0.09	0.16	0.38	2.15
Firmicutes	*Clostridia*	*Clostridiales*	*Clostridiaceae*	*SMB53*	0.2 ± 0.05	0.08 ± 0.04	0.12	0.2	0.43	0.22 ± 0.14	0.42 ± 0.19	0.43	0.58	1.88
Firmicutes	*Clostridia*	*Clostridiales*	*Clostridiaceae*	-	0.07 ± 0.01	0.03 ± 0.02	0.12	0.21	0.42	0.11 ± 0.04	0.11 ± 0.05	0.93	0.93	1.06
Firmicutes	*Bacilli*	*Lactobacillales*	*Lactobacillaceae*	*Lactobacillus*	4.42 ± 1.23	9.81 ± 3.32	0.17	0.26	2.22	4.95 ± 2.17	16.25 ± 5.8	0.11	0.27	3.29
Bacteroidetes	*Bacteroidia*	*Bacteroidales*	*Rikenellaceae*	-	2.25 ± 0.46	1.41 ± 0.37	0.18	0.26	0.63	2.17 ± 0.26	0.83 ± 0.13	**<0.01**	**0.03**	0.38
Firmicutes	*Clostridia*	*Clostridiales*	*Ruminococcaceae*	-	4.79 ± 0.73	3.21 ± 0.94	0.21	0.29	0.67	5.2 ± 0.48	3.53 ± 1.42	0.3	0.56	0.68
Firmicutes	*Clostridia*	*Clostridiales*	*Dehalobacteriaceae*	*Dehalobacterium*	0.31 ± 0.05	0.19 ± 0.07	0.22	0.29	0.62	0.16 ± 0.02	0.05 ± 0.02	**0.01**	**0.05**	0.34
Firmicutes	*Clostridia*	*Clostridiales*	*Lachnospiraceae*	*Roseburia*	0.24 ± 0.09	1.79 ± 1.25	0.26	0.33	7.56	0.21 ± 0.12	0.16 ± 0.1	0.76	0.89	0.77
Firmicutes	*Clostridia*	*Clostridiales*	*Lachnospiraceae*	*Coprococcus*	3.04 ± 0.74	4.46 ± 1.04	0.29	0.35	1.47	2.08 ± 0.5	3.35 ± 1.27	0.38	0.58	1.61
Firmicutes	*Erysipelotrichi*	*Erysipelotrichales*	*Erysipelotrichaceae*	*Allobaculum*	0	1.71 ± 1.65	0.34	0.4	3220.5	0	0.3 ± 0.23	0.24	0.48	1024.8
Firmicutes	*Clostridia*	*Clostridiales*	*Lachnospiraceae*	-	9.04 ± 2.13	7.17 ± 1.01	0.45	0.5	0.79	6.36 ± 1.38	6.04 ± 1.78	0.89	0.93	0.95
Firmicutes	*Clostridia*	*Clostridiales*	*Mogibacteriaceae*	-	0.12 ± 0.03	0.15 ± 0.03	0.49	0.53	1.22	0.22 ± 0.06	0.1 ± 0.03	0.1	0.27	0.46
Firmicutes	*Clostridia*	*Clostridiales*	*Ruminococcaceae*	*Ruminococcus*	1.45 ± 0.3	1.22 ± 0.39	0.65	0.67	0.84	1.08 ± 0.18	0.79 ± 0.25	0.36	0.58	0.73
Firmicutes	*Clostridia*	*Clostridiales*	*Clostridiaceae*	*Clostridium*	0.14 ± 0.11	0.18 ± 0.09	0.81	0.81	1.25	0.44 ± 0.21	0.9 ± 0.42	0.36	0.58	2.04

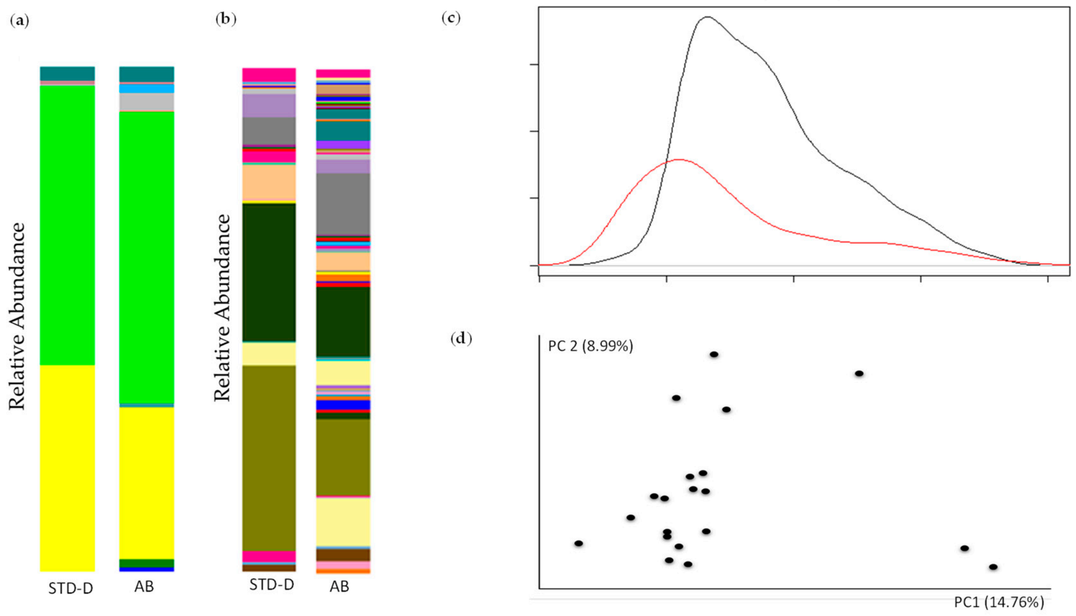

Figure S2. Microbiota analysis after the AB treatment compared with a control group (STD-D group). (**a**) Phyla relative abundance, (**b**) Genus relative abundance, (**c**) Density plot of OTU vs genus: STD-D (black line) and after treatment (red line) with significant differences in area (p = 0.04), (**d**) Analysis of beta diversity represented by scores after the AB treatment with PCoA (unweighted unifrac).

Table S2. Biometric parameters, plasma parameters and liver biochemistry of transplant model. Data are presented as the mean ± S.E.M. (n=7). The statistical comparisons among groups were conducted using Student's t test, the statistically significant p-values ($p < 0.05$) are highlighted in bold. RWAT, retroperitoneal white adipose tissue; MWAT, mesenteric white adipose tissue; TG, triglycerides; TC, total cholesterol; NEFAs, non-esterified fatty acids.

		**Mean ± S.E.M.**			**P-values**		
		**CNT-R**	**STD-R**	**CAF-R**	**CNT-R vs STD-R**	**CNT-R vs CAF-R**	**STD-R vs CAF-R**
**Biometric parameters**	Initial body weight (g)	334.55 ± 17.02	341.55 ± 9.62	341.13 ± 12.16	0.37	0.42	0.94
	Final body weight (g)	415.24 ± 10.86	407.10 ± 7.56	420.47 ± 11.98	0.55	0.74	0.34
	Food intake (g)	24.06 ± 0.80	23.26 ± 1.06	23.97 ± 1.01	0.56	0.95	0.64
	RWAT weight (g)	6.73 ± 0.96	7.14 ± 0.90	9.70 ± 1.70	0.76	0.17	0.22
	MWAT weight (g)	4.40 ± 0.46	4.04 ± 0.26	5.16 ± 0.52	0.5	0.29	0.09
	Muscle weigth (g)	2.49 ± 0.08	2.51 ± 0.06	2.49 ± 0.08	0.86	0.97	0.89
	Liver weight (g)	11.61 ± 0.50	11.57 ± 0.52	11.92 ± 0.59	0.96	0.69	0.66
	CEC weight (g)	8.43 ± 0.63	5.23 ± 0.26	5.33 ± 0.38	**0.002**	**0.002**	0.82
Plasma	Glucose (mM)	74.20 ± 2.45	77.88 ± 5.81	74.46 ± 1.88	0.58	0.93	0.59
	TG (mM)	77.06 ± 4.07	80.91 ± 14.01	117.30 ± 17.12	0.8	0.07	0.13
	TC (mM)	42.34 ± 2.62	44.81 ± 9	68.19 ± 11	0.8	0.07	0.13
	NEFAs (mM)	0.35 ± 0.02	0.37 ± 0.02	0.43 ± 0.03	0.69	**0.04**	0.11
Liver	Total lipids (mg/g)	40.98 ± 3.54	38.57 ± 2.20	28.99 ± 2.22	0.58	**0.02**	**0.01**
	TC (mg/g)	1.83 ± 0.08	1.98 ± 0.11	1.83 ± 0.08	0.28	0.97	0.27
	Phospholipids (mg/g)	12.25 ± 0.67	12.85 ± 0.60	10.67 ± 0.67	0.52	0.12	**0.03**
	TG (mg/g)	4.10 ± 0.27	4.22 ± 0.23	4.07 ± 0.34	0.75	0.94	0.72

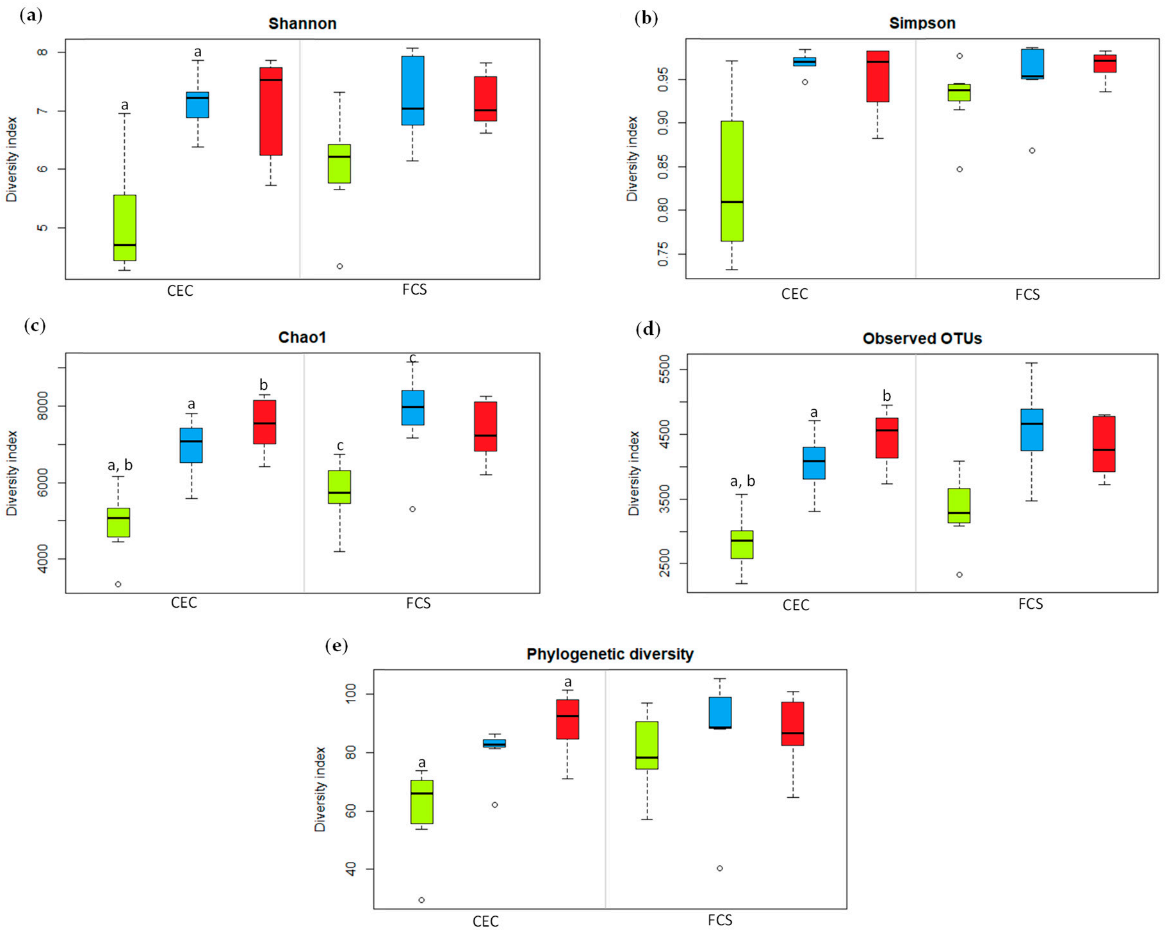

Figure S3. Characterization of the alpha diversity indices derived from the QIIME command α rarefraction after the microbiota transplant in different metagenomic biofluids (CEC and FCS): (**a**) Shannon index, (**b**) Simpson index, (**c**) Chao1, (**d**) Observed OTUs, (**e**) Phylogenetic diversity. Different lowercase letters a and b indicate significant ($p < 0.05$) changes between diets. Green, CNT-R; blue, STD-R; red, CAF-R.

Table S3. Summary of metagenomics in the CNT-R and the STD-R groups in CEC and FCS focusing on taxonomic data. Taxonomic data presented as the mean ± S.E.M. (n=7) per group shorted by q-value of CEC. The summary of univariant analysis is shown including p-value, q-value and FC, the statistically significant p-values and q-values (< 0.05) are highlighted in bold.

					**CEC**					**FCS**				
**Phylum**	**Class**	**Order**	**Family**	**Genus**	**CNT-R (%)**	**STD-R (%)**	***p*-value**	***q*-value**	**FC**	**CNT-R (%)**	**STD-R (%)**	***p*-value**	***q*-value**	**FC**
Firmicutes	*Clostridia*	*Clostridiales*	*Ruminococcaceae*	*Ruminococcus*	0.68 ± 0.26	2.1 ± 0.79	**<0.01**	**<0.01**	3.09	2.41 ± 0.91	2.63 ± 1	0.85	0.92	1.09
Firmicutes	*Clostridia*	*Clostridiales*	*Lachnospiraceae*	-	3.27 ± 1.24	2.85 ± 1.08	**<0.01**	**0.01**	0.87	18.3 ± 6.91	8.59 ± 3.25	0.15	0.3	0.47
Firmicutes	*Clostridia*	*Clostridiales*	*Ruminococcaceae*	*Oscillospira*	2.51 ± 0.95	2.31 ± 0.87	**<0.01**	**0.01**	0.92	7.51 ± 2.84	4.58 ± 1.73	0.72	0.81	0.61
Firmicutes	*Clostridia*	*Clostridiales*	*Lachnospiraceae*	*Coprococcus*	1.05 ± 0.4	1.22 ± 0.46	**<0.01**	**0.02**	1.15	3.77 ± 1.43	2.72 ± 1.03	0.25	0.38	0.72
Actinobacteria	*Coriobacteriia*	*Coriobacteriales*	*Coriobacteriaceae*	*Adlercreutzia*	0.09 ± 0.04	0.09 ± 0.03	**0.01**	0.06	0.99	0.02 ± 0.01	0.06 ± 0.02	**<0.01**	**0.02**	3.33
Firmicutes	*Clostridia*	*Clostridiales*	*Lachnospiraceae*	*Roseburia*	0.07 ± 0.03	0.03 ± 0.01	**0.02**	0.08	0.47	0.38 ± 0.14	0.18 ± 0.07	0.39	0.54	0.47
Firmicutes	*Clostridia*	*Clostridiales*	*Peptococcaceae*	*rc4-4*	0.07 ± 0.03	0.17 ± 0.06	**0.02**	0.08	2.46	0.18 ± 0.07	0.47 ± 0.18	0.43	0.57	2.63
Verrucomicrobia	*Verrucomicrobiae*	*Verrucomicrobiales*	*Verrucomicrobiaceae*	*Akkermansia*	8.46 ± 3.2	12.9 ± 4.88	**0.03**	0.08	1.53	0.78 ± 0.3	2.97 ± 1.12	0.98	0.98	3.79
Bacteroidetes	*Bacteroidia*	*Bacteroidales*	*Bacteroidaceae*	*Bacteroides*	0.43 ± 0.16	5.07 ± 1.92	**0.05**	0.14	11.9	0.09 ± 0.03	0.42 ± 0.16	**0.02**	0.12	4.65
Proteobacteria	*Gammaproteobacteria*	*Enterobacteriales*	*Enterobacteriaceae*	-	0.62 ± 0.24	0.79 ± 0.3	0.06	0.16	1.26	0.21 ± 0.08	0.22 ± 0.08	0.93	0.97	1.06
Firmicutes	*Clostridia*	*Clostridiales*	*Ruminococcaceae*	-	2.52 ± 0.95	2.35 ± 0.89	0.11	0.24	0.94	4.54 ± 1.72	6.7 ± 2.53	0.63	0.75	1.48
Bacteroidetes	*Bacteroidia*	*Bacteroidales*	*S24-7*	-	17.7 ± 6.68	38.3 ± 14.5	0.15	0.31	2.17	6.96 ± 2.63	41.1 ± 15.5	0.03	0.17	5.91
Firmicutes	*Clostridia*	*Clostridiales*	*Lachnospiraceae*	*Anaerostipes*	0.03 ± 0.01	0.13 ± 0.05	0.26	0.5	3.71	0.07 ± 0.03	0.23 ± 0.09	0.21	0.37	3.37
Actinobacteria	*Actinobacteria*	*Bifidobacteriales*	*Bifidobacteriaceae*	*Bifidobacterium*	0.02 ± 0.01	0.25 ± 0.09	0.31	0.51	12.4	-	0.57 ± 0.21	**<0.01**	**<0.01**	133.67
Firmicutes	*Clostridia*	*Clostridiales*	*Clostridiaceae*	*SMB53*	0.07 ± 0.03	0.31 ± 0.12	0.29	0.51	4.49	0.19 ± 0.07	0.41 ± 0.16	0.12	0.26	2.19
Firmicutes	*Clostridia*	*Clostridiales*	*Lachnospiraceae*	*[Ruminococcus]*	1.45 ± 0.55	1.11 ± 0.42	0.33	0.51	0.76	0.91 ± 0.34	0.42 ± 0.16	0.15	0.3	0.46
Firmicutes	*Bacilli*	*Turicibacterales*	*Turicibacteraceae*	*Turicibacter*	0.06 ± 0.02	0.59 ± 0.22	0.45	0.66	9.6	0.04 ± 0.02	0.41 ± 0.15	0.06	0.18	9.86
Firmicutes	*Clostridia*	*Clostridiales*	*[Mogibacteriaceae]*	-	0.12 ± 0.04	0.2 ± 0.08	0.48	0.66	1.68	0.08 ± 0.03	0.2 ± 0.07	0.07	0.18	2.41
Firmicutes	*Bacilli*	*Lactobacillales*	*Lactobacillaceae*	*Lactobacillus*	3.46 ± 1.31	11.4 ± 4.33	0.59	0.7	3.3	2.8 ± 1.06	6.27 ± 2.37	**0.04**	0.17	2.24
Firmicutes	*Clostridia*	*Clostridiales*	-	-	55.4 ± 20.9	12.4 ± 4.68	0.55	0.7	0.22	48.8 ± 18.5	17 ± 6.42	0.06	0.18	0.35
Firmicutes	*Clostridia*	*Clostridiales*	*Clostridiaceae*	*Clostridium*	0.23 ± 0.09	0.51 ± 0.19	0.57	0.7	2.19	0.16 ± 0.06	0.73 ± 0.28	0.12	0.26	4.72
Firmicutes	*Clostridia*	*Clostridiales*	*Lachnospiraceae*	*Blautia*	0.34 ± 0.13	1.38 ± 0.52	0.65	0.74	4.13	0.28 ± 0.11	0.13 ± 0.05	0.24	0.38	0.46
Bacteroidetes	*Bacteroidia*	*Bacteroidales*	*Rikenellaceae*	-	0.69 ± 0.26	1.13 ± 0.43	0.79	0.86	1.64	0.76 ± 0.29	1.25 ± 0.47	**0.02**	0.12	1.64
Firmicutes	*Clostridia*	*Clostridiales*	*Peptostreptococcaceae*	-	0.16 ± 0.06	0.3 ± 0.11	0.91	0.95	1.9	0.17 ± 0.06	0.29 ± 0.11	0.52	0.65	1.74
Firmicutes	*Clostridia*	*Clostridiales*	*Lachnospiraceae*	*Dorea*	0.07 ± 0.02	0.18 ± 0.07	0.99	0.99	2.73	0.07 ± 0.02	0.09 ± 0.03	0.28	0.4	1.39

Table S4. Summary of metagenomics in the CNT-R and the CAF-R groups in CEC and FCS focusing on taxonomic data. Taxonomic data presented as the mean ± S.E.M. (n=7) per group shorted by q-value of CEC. The summary of univariant analysis is shown including p-value, q-value and FC, the statistically significant p-values and q-values (< 0.05) are highlighted in bold.

					**CEC**					**FCS**				
**Phylum**	**Class**	**Order**	**Family**	**Genus**	**CNT-R (%)**	**CAF-R (%)**	***p*-value**	***q*-value**	**FC**	**CNT-R (%)**	**CAF-R (%)**	***p*-value**	***q*-value**	**FC**
Firmicutes	*Clostridia*	*Clostridiales*	*Ruminococcaceae*	*Oscillospira*	2.51 ± 0.95	7.45 ± 3.04	**<0.01**	**0.01**	2.97	2.31 ± 0.87	4.63 ± 1.75	**<0.01**	0.04	2
Firmicutes	*Clostridia*	*Clostridiales*	*Lachnospiraceae*	*Coprococcus*	1.05 ± 0.4	3.01 ± 1.23	**<0.01**	**0.04**	2.85	1.22 ± 0.46	1.79 ± 0.68	0.23	0.53	1.47
Firmicutes	*Clostridia*	*Clostridiales*	*Ruminococcaceae*	*Ruminococcus*	0.68 ± 0.26	2.24 ± 0.92	**<0.01**	**0.04**	3.3	2.1 ± 0.79	2.59 ± 0.98	0.46	0.6	1.23
Firmicutes	*Clostridia*	*Clostridiales*	*Lachnospiraceae*	-	3.27 ± 1.24	9.31 ± 3.8	**0.01**	0.06	2.85	2.85 ± 1.08	3.26 ± 1.23	0.59	0.72	1.14
Actinobacteria	*Coriobacteriia*	*Coriobacteriales*	*Coriobacteriaceae*	-	0.02 ± 0.01	-	**0.02**	0.1	0.21	0.02 ± 0.01	0.01 ± 0	0.29	0.55	0.54
Verrucomicrobia	*Verrucomicrobiae*	*Verrucomicrobiales*	*Verrucomicrobiaceae*	*Akkermansia*	8.46 ± 3.2	0.73 ± 0.3	**0.02**	0.1	0.09	12.9 ± 4.88	2.23 ± 0.84	0.03	0.24	0.17
Actinobacteria	*Coriobacteriia*	*Coriobacteriales*	*Coriobacteriaceae*	*Adlercreutzia*	0.09 ± 0.04	0.03 ± 0.01	**0.03**	0.11	0.37	0.09 ± 0.03	0.1 ± 0.04	0.79	0.82	1.11
Firmicutes	*Erysipelotrichi*	*Erysipelotrichales*	*Erysipelotrichaceae*	-	0.05 ± 0.02	0.02 ± 0.01	**0.03**	0.11	0.28	0.21 ± 0.08	0.09 ± 0.04	0.2	0.53	0.45
Firmicutes	*Clostridia*	*Clostridiales*	*Lachnospiraceae*	*Dorea*	0.07 ± 0.02	0.26 ± 0.11	0.1	0.3	4.01	0.18 ± 0.07	0.29 ± 0.11	0.22	0.53	1.61
Firmicutes	*Clostridia*	*Clostridiales*	*Peptococcaceae*	*rc4-4*	0.07 ± 0.03	0.14 ± 0.06	0.13	0.36	2.11	0.17 ± 0.06	0.27 ± 0.1	0.27	0.54	1.61
Firmicutes	*Clostridia*	*Clostridiales*	*Lachnospiraceae*	*[Ruminococcus]*	1.45 ± 0.55	0.64 ± 0.26	0.16	0.41	0.44	1.11 ± 0.42	0.42 ± 0.16	0.01	0.17	0.38
Bacteroidetes	*Bacteroidia*	*Bacteroidales*	*S24-7*	-	17.7 ± 6.68	7.94 ± 3.24	0.2	0.44	0.45	38.4 ± 14.5	41.1 ± 15.5	0.69	0.75	1.07
Tenericutes	*Mollicutes*	*RF39*	-	-	0.03 ± 0.01	0.39 ± 0.16	0.2	0.44	15.41	0.12 ± 0.04	0.58 ± 0.22	0.14	0.5	5.02
Proteobacteria	*Gammaproteobacteria*	*Enterobacteriales*	*Enterobacteriaceae*	-	0.62 ± 0.24	0.33 ± 0.14	0.24	0.47	0.54	0.79 ± 0.3	0.52 ± 0.2	0.34	0.58	0.66
Bacteroidetes	*Bacteroidia*	*Bacteroidales*	*Porphyromonadaceae*	*Parabacteroides*	0.05 ± 0.02	0.13 ± 0.06	0.26	0.49	2.65	0.15 ± 0.06	0.69 ± 0.26	0.02	0.22	4.48
Firmicutes	*Clostridia*	*Clostridiales*	*Ruminococcaceae*	-	2.52 ± 0.95	3.65 ± 1.49	0.28	0.5	1.45	2.35 ± 0.89	4.52 ± 1.71	0.05	0.25	1.92
Firmicutes	*Clostridia*	*Clostridiales*	*Lachnospiraceae*	*Blautia*	0.34 ± 0.13	0.21 ± 0.09	0.38	0.59	0.63	1.38 ± 0.52	0.13 ± 0.05	0.12	0.48	0.09
Firmicutes	*Clostridia*	*Clostridiales*	*Peptostreptococcaceae*	-	0.16 ± 0.06	0.11 ± 0.04	0.36	0.59	0.66	0.3 ± 0.11	0.68 ± 0.26	0.25	0.53	2.27
Firmicutes	*Clostridia*	*Clostridiales*	*Clostridiaceae*	*SMB53*	0.07 ± 0.03	0.05 ± 0.02	0.51	0.75	0.76	0.31 ± 0.12	0.55 ± 0.21	0.42	0.58	1.78
Bacteroidetes	*Bacteroidia*	*Bacteroidales*	*Bacteroidaceae*	*Bacteroides*	0.43 ± 0.16	0.33 ± 0.13	0.66	0.84	0.77	5.07 ± 1.92	1 ± 0.38	0.24	0.53	0.2
Firmicutes	*Bacilli*	*Turicibacterales*	*Turicibacteraceae*	*Turicibacter*	0.06 ± 0.02	0.04 ± 0.02	0.6	0.84	0.67	0.59 ± 0.22	1.22 ± 0.46	0.35	0.58	2.08
Firmicutes	*Clostridia*	*Clostridiales*	*Lachnospiraceae*	*Anaerostipes*	0.03 ± 0.01	0.03 ± 0.01	0.65	0.84	0.77	0.13 ± 0.05	0.16 ± 0.06	0.67	0.75	1.25
Firmicutes	*Clostridia*	*Clostridiales*	*Clostridiaceae*	*Clostridium*	0.23 ± 0.09	0.18 ± 0.07	0.72	0.88	0.78	0.51 ± 0.19	1.25 ± 0.47	0.05	0.25	2.46
Firmicutes	*Bacilli*	*Lactobacillales*	*Lactobacillaceae*	*Lactobacillus*	3.46 ± 1.31	3.16 ± 1.29	0.82	0.95	0.91	11.4 ± 4.3	12.7 ± 4.81	0.69	0.75	1.11
Firmicutes	*Clostridia*	*Clostridiales*	-	-	55.3 ± 20.9	57.4 ± 23.4	0.86	0.96	1.04	12.3 ± 4.6	16.3 ± 6.15	0.37	0.58	1.31
Bacteroidetes	*Bacteroidia*	*Bacteroidales*	*Rikenellaceae*	-	0.69 ± 0.26	0.72 ± 0.3	0.9	0.97	1.05	1.13 ± 0.43	1.14 ± 0.43	0.98	0.98	1.01
Actinobacteria	*Actinobacteria*	*Bifidobacteriales*	*Bifidobacteriaceae*	*Bifidobacterium*	0.02 ± 0.01	0.02 ± 0.01	0.98	0.98	0.97	0.25 ± 0.09	0.44 ± 0.17	0.4	0.58	1.76

Firmicutes	*Clostridia*	*Clostridiales*	*[Mogibacteriaceae]*	-	0.12 ± 0.04	0.12 ± 0.05	0.95	0.98	1.03	0.2 ± 0.08	0.27 ± 0.1	0.47	0.6	1.35

Table S5. Summary of metagenomics in the STD-R and the CAF-R groups in CEC and FCS focusing on taxonomic data. Taxonomic data presented as the mean ± S.E.M. (n=7) per group shorted by q-value of CEC. The summary of univariant analysis is shown including p-value, q-value and FC; the statistically significant p-values and q-values (< 0.05) are highlighted in bold.

					**CEC**					**FCS**				
**Phylum**	**Class**	**Order**	**Family**	**Genus**	**STD-R (%)**	**CAF-R (%)**	***p*-value**	***q*-value**	**FC**	**STD-R (%)**	**CAF-R (%)**	***p*-value**	***q*-value**	**FC**
Firmicutes	*Clostridia*	*Clostridiales*	*Lachnospiraceae*	-	18.29 ± 6.91	9.31 ± 3.8	**0.02**	0.46	0.51	8.59 ± 3.25	3.26 ± 1.23	**0.01**	0.1	0.38
Bacteroidetes	*Bacteroidia*	*Bacteroidales*	*Porphyromonadaceae*	*Parabacteroides*	0.03 ± 0.01	0.13 ± 0.06	0.08	0.65	4.69	0.16 ± 0.06	0.69 ± 0.26	**0.01**	0.1	4.28
Firmicutes	*Clostridia*	*Clostridiales*	*Lachnospiraceae*	*Dorea*	0.07 ± 0.02	0.26 ± 0.11	0.09	0.65	3.99	0.09 ± 0.03	0.29 ± 0.11	**0.02**	0.1	3.16
Firmicutes	*Clostridia*	*Clostridiales*	*Lachnospiraceae*	*Roseburia*	0.38 ± 0.14	0.75 ± 0.31	0.09	0.65	1.97	0.18 ± 0.07	0.29 ± 0.11	0.37	0.61	1.6
Bacteroidetes	*Bacteroidia*	*Bacteroidales*	*Bacteroidaceae*	*Bacteroides*	0.09 ± 0.03	0.33 ± 0.13	0.17	0.67	3.65	0.42 ± 0.16	1 ± 0.38	0.06	0.23	2.41
Firmicutes	*Clostridia*	*Clostridiales*	*[Mogibacteriaceae]*	-	0.08 ± 0.03	0.12 ± 0.05	0.24	0.67	1.5	0.2 ± 0.07	0.27 ± 0.1	0.2	0.44	1.36
Firmicutes	*Clostridia*	*Clostridiales*	*Peptostreptococcaceae*	-	0.17 ± 0.06	0.11 ± 0.04	0.2	0.67	0.64	0.29 ± 0.11	0.68 ± 0.26	0.22	0.44	2.38
Tenericutes	*Mollicutes*	*RF39*	-	-	0.07 ± 0.03	0.39 ± 0.16	0.26	0.67	5.33	0.26 ± 0.1	0.58 ± 0.22	0.29	0.51	2.26
Firmicutes	*Clostridia*	*Clostridiales*	*Lachnospiraceae*	*Anaerostipes*	0.07 ± 0.03	0.03 ± 0.01	0.18	0.67	0.38	0.23 ± 0.09	0.16 ± 0.06	0.46	0.71	0.68
Firmicutes	*Clostridia*	*Clostridiales*	*Clostridiaceae*	*SMB53*	0.19 ± 0.07	0.05 ± 0.02	0.25	0.67	0.28	0.41 ± 0.16	0.55 ± 0.21	0.62	0.86	1.32
Firmicutes	*Clostridia*	*Clostridiales*	-	-	48.8 ± 18.45	57.49 ± 23.47	0.25	0.67	1.18	16.99 ± 6.42	16.28 ± 6.15	0.81	0.98	0.96
Firmicutes	*Clostridia*	*Clostridiales*	*Lachnospiraceae*	*[Ruminococcus]*	0.91 ± 0.34	0.64 ± 0.26	0.3	0.69	0.71	0.42 ± 0.16	0.42 ± 0.16	0.99	0.99	1
Firmicutes	*Clostridia*	*Clostridiales*	*Lachnospiraceae*	*Coprococcus*	3.77 ± 1.43	3.01 ± 1.23	0.36	0.77	0.8	2.72 ± 1.03	1.79 ± 0.68	0.18	0.44	0.66
Firmicutes	*Clostridia*	*Clostridiales*	*Ruminococcaceae*	-	4.54 ± 1.72	3.65 ± 1.49	0.44	0.78	0.81	6.7 ± 2.53	4.52 ± 1.71	0.08	0.28	0.67
Proteobacteria	*Gammaproteobacteria*	*Enterobacteriales*	*Enterobacteriaceae*	-	0.21 ± 0.08	0.33 ± 0.14	0.44	0.78	1.6	0.22 ± 0.08	0.52 ± 0.2	0.12	0.33	2.35
Actinobacteria	*Actinobacteria*	*Bifidobacteriales*	*Bifidobacteriaceae*	*Bifidobacterium*	-	0.02 ± 0.01	0.39	0.78	4.63	0.57 ± 0.21	0.44 ± 0.17	0.65	0.86	0.78
Firmicutes	*Clostridia*	*Clostridiales*	*Peptococcaceae*	*rc4-4*	0.18 ± 0.07	0.14 ± 0.06	0.49	0.79	0.79	0.47 ± 0.18	0.27 ± 0.1	**0.02**	0.1	0.56
Firmicutes	*Clostridia*	*Clostridiales*	*Clostridiaceae*	-	0.04 ± 0.01	0.07 ± 0.03	0.53	0.79	2.05	0.19 ± 0.07	0.46 ± 0.17	0.24	0.45	2.44
Firmicutes	*Clostridia*	*Clostridiales*	*Lachnospiraceae*	*Blautia*	0.28 ± 0.11	0.21 ± 0.09	0.51	0.79	0.76	0.13 ± 0.05	0.13 ± 0.05	0.94	0.99	0.98
Firmicutes	*Clostridia*	*Clostridiales*	*Dehalobacteriaceae*	*Dehalobacterium*	0.19 ± 0.07	0.21 ± 0.09	0.63	0.88	1.09	0.09 ± 0.04	0.05 ± 0.02	**0.02**	0.1	0.52
Firmicutes	*Bacilli*	*Lactobacillales*	*Lactobacillaceae*	*Lactobacillus*	2.8 ± 1.06	3.16 ± 1.29	0.73	0.9	1.13	6.27 ± 2.37	12.73 ± 4.81	**0.01**	0.1	2.03
Firmicutes	*Clostridia*	*Clostridiales*	*Ruminococcaceae*	*Ruminococcus*	2.41 ± 0.91	2.24 ± 0.92	0.74	0.9	0.93	2.63 ± 1	2.59 ± 0.98	0.94	0.99	0.98
Bacteroidetes	*Bacteroidia*	*Bacteroidales*	*S24-7*	-	6.96 ± 2.63	7.94 ± 3.24	0.7	0.9	1.14	41.15 ± 15.55	41.04 ± 15.51	0.98	0.99	1
Firmicutes	*Clostridia*	*Clostridiales*	*Clostridiaceae*	*Clostridium*	0.16 ± 0.06	0.18 ± 0.07	0.8	0.93	1.17	0.73 ± 0.28	1.25 ± 0.47	0.12	0.33	1.7

Verrucomicrobia	*Verrucomicrobiae*	*Verrucomicrobiales*	*Verrucomicrobiaceae*	*Akkermansia*	0.78 ± 0.3	0.73 ± 0.3	0.9	0.97	0.93	2.97 ± 1.12	2.23 ± 0.84	0.57	0.83	0.75
Bacteroidetes	*Bacteroidia*	*Bacteroidales*	*Rikenellaceae*	-	0.76 ± 0.29	0.72 ± 0.3	0.88	0.97	0.95	1.25 ± 0.47	1.14 ± 0.43	0.7	0.89	0.91
Firmicutes	*Bacilli*	*Turicibacterales*	*Turicibacteraceae*	*Turicibacter*	0.04 ± 0.02	0.04 ± 0.02	0.99	0.99	1.01	0.41 ± 0.15	1.22 ± 0.46	0.21	0.44	3.02
Firmicutes	*Clostridia*	*Clostridiales*	*Ruminococcaceae*	*Oscillospira*	7.51 ± 2.84	7.45 ± 3.04	0.96	0.99	0.99	4.58 ± 1.73	4.63 ± 1.75	0.96	0.99	1.01

Table S6. Statistical analysis of plasma metabolites in the STD-R and the CAF-R groups. 139 metabolites presented as the mean ± S.E.M. per group shorted by p-value. The summary of univariant analysis is shown including p-value, q-value and FC; the statistically significant p-values and q-values (< 0.05) are highlighted in bold. DG, diacylglycerol; LPC, lysophospholipid; PC, phosphatidylcholine; ChoE, cholesterol ester; SM, sphingomyelin; TG, triglyceride; PE, phosphatidylethanolamine.

Metabolite	STD-R	CAF-R	*p*-value	*q*-value	FC
DG 34:2	0.42 ± 0.04	0.72 ± 0.04	**<0.01**	**0.05**	0.09
DG 34:3	0.08 ± 0.02	0.17 ± 0.02	**0.01**	0.58	0.23
DG 36:2	0.9 ± 0.09	1.31 ± 0.12	**0.01**	0.64	0.14
DG 36:4	0.7 ± 0.07	0.96 ± 0.06	**0.02**	0.72	0.09
LPC 20:0	0.35 ± 0.02	0.3 ± 0.01	**0.03**	0.74	0.03
DG 34:1	0.78 ± 0.05	1.02 ± 0.07	**0.03**	0.80	0.10
PC 31:0	0.04 ± 0	0.03 ± 0	**0.04**	0.88	0.12
glyceric acid	1.22 ± 0.07	1.42 ± 0.05	0.06	0.89	0.04
PC 42:4 e	0.01 ± 0	0.01 ± 0	0.07	0.89	0.10
PC 36:3 e	0.06 ± 0	0.05 ± 0	0.07	0.89	0.05
ChoE (16:0)	2.57 ± 0.22	2.08 ± 0.15	0.10	0.89	0.06
oleic acid	1.46 ± 0.1	1.72 ± 0.13	0.12	0.89	0.09
ChoE (18:2)	20.18 ± 1.95	16.1 ± 1.27	0.12	0.89	0.06
PC 35:2	0.42 ± 0.04	0.34 ± 0.03	0.13	0.89	0.06
ribose	4.37 ± 0.35	3.28 ± 0.51	0.13	0.89	0.12
Fumaric acid	0.71 ± 0.07	0.88 ± 0.08	0.14	0.89	0.12
ChoE (18:1)	3.04 ± 0.29	2.5 ± 0.17	0.15	0.89	0.06
LPC 15:0	0.98 ± 0.06	0.85 ± 0.06	0.15	0.89	0.06
SM 42:3	5.67 ± 0.45	4.87 ± 0.23	0.15	0.89	0.04
TG 52:3	39.46 ± 10.4	62.08 ± 11.23	0.16	0.89	0.28
aconitic acid	0.01 ± 0	0.01 ± 0	0.16	0.89	0.10
TG 54:6	13.27 ± 1.87	19.03 ± 3.23	0.18	0.89	0.24
Threonic acid	1.68 ± 0.27	2.13 ± 0.15	0.19	0.89	0.09
ChoE (18:0)	0.15 ± 0.01	0.12 ± 0.01	0.19	0.89	0.07
PC 33:0	0.04 ± 0	0.03 ± 0	0.19	0.89	0.07
SM 43:1	1.34 ± 0.1	1.14 ± 0.1	0.20	0.89	0.07
TG 54:4	11.42 ± 3.18	17.76 ± 3.49	0.20	0.89	0.31
Cholesterol	0.51 ± 0.1	0.37 ± 0.02	0.20	0.89	0.04
TG 48:0	1.24 ± 0.19	1.88 ± 0.43	0.23	0.89	0.35
TG 50:0	0.37 ± 0.04	0.5 ± 0.08	0.23	0.89	0.22
SM 35:1	0.18 ± 0.01	0.16 ± 0.01	0.24	0.89	0.05
PC 40:5	0.33 ± 0.07	0.53 ± 0.14	0.24	0.89	0.42
TG 54:3	4.01 ± 1.08	5.87 ± 1.15	0.26	0.89	0.29
TG 54:2	0.66 ± 0.16	0.96 ± 0.19	0.27	0.89	0.29
Glucose-6-phosphate	0.17 ± 0.02	0.14 ± 0.02	0.27	0.89	0.11
TG 54:7	4.94 ± 1.19	6.91 ± 1.18	0.27	0.89	0.24
SM 41:2	0.65 ± 0.03	0.69 ± 0.03	0.29	0.89	0.05
TG 52:1	0.63 ± 0.14	0.91 ± 0.2	0.29	0.89	0.32
malic acid	0.38 ± 0.03	0.44 ± 0.04	0.29	0.89	0.11
TG 52:5	7.38 ± 2.04	10.47 ± 1.92	0.29	0.89	0.26
TG 52:2	13.12 ± 5.39	22.41 ± 6.39	0.30	0.89	0.49
alpha-ketoglutarate	1.27 ± 0.07	1.43 ± 0.13	0.30	0.89	0.10

LPC 16:0	83.94 ± 2.63	79.81 ± 2.68	0.30	0.89	0.03
TG 48:1	1.82 ± 0.51	3.29 ± 1.13	0.30	0.89	0.62
PE 38:5 e	1.91 ± 0.32	2.78 ± 0.67	0.31	0.89	0.35
TG 50:1	3.77 ± 1.33	6.83 ± 2.45	0.32	0.89	0.65
PC 38:3	0.97 ± 0.15	1.21 ± 0.17	0.33	0.89	0.17
TG 50:2	13.23 ± 4.96	22.99 ± 7.66	0.33	0.89	0.58
TG 52:6	1.13 ± 0.36	1.61 ± 0.31	0.33	0.89	0.28
LPC 16:0 e	0.56 ± 0.04	0.51 ± 0.02	0.35	0.89	0.04
Glucose	0.77 ± 0.04	0.72 ± 0.03	0.35	0.89	0.04
ChoE (17:0)	0.16 ± 0.02	0.14 ± 0.01	0.35	0.89	0.08
TG 50:3	7.94 ± 3.58	13.26 ± 4.12	0.36	0.89	0.52
SM 40:2	0.69 ± 0.08	0.77 ± 0.04	0.38	0.89	0.05
PC 32:0	0.7 ± 0.04	0.64 ± 0.05	0.38	0.89	0.07
TG 46:1	0.63 ± 0.08	0.8 ± 0.15	0.38	0.89	0.25
citric acid	3.9 ± 0.13	3.67 ± 0.19	0.38	0.89	0.05
SM 32:1	0.28 ± 0.02	0.32 ± 0.03	0.41	0.89	0.11
TG 50:4	1.86 ± 0.75	2.73 ± 0.69	0.41	0.89	0.37
PC 36:0	0.09 ± 0.01	0.11 ± 0.02	0.42	0.89	0.20
TG 46:0	0.85 ± 0.09	0.96 ± 0.09	0.43	0.89	0.11
ChoE (16:1)	0.77 ± 0.13	0.92 ± 0.14	0.43	0.89	0.18
TG 51:2	0.83 ± 0.26	1.16 ± 0.3	0.43	0.89	0.36
TG 48:2	1.9 ± 0.77	3.19 ± 1.29	0.43	0.89	0.68
PC 38:6 e	0.07 ± 0.01	0.06 ± 0.01	0.44	0.89	0.09
Urea	2.76 ± 0.18	2.58 ± 0.15	0.45	0.89	0.05
PE 36:4	4.09 ± 0.7	4.78 ± 0.6	0.45	0.89	0.15
ChoE (18:3)	1.55 ± 0.11	1.4 ± 0.15	0.46	0.89	0.10
PC 38:4 e	0.06 ± 0.01	0.06 ± 0	0.47	0.89	0.08
isoleucine	0.3 ± 0.16	0.69 ± 0.45	0.49	0.89	1.49
SM 34:2	1.58 ± 0.07	1.7 ± 0.14	0.49	0.89	0.09
leucine	0.11 ± 0.06	0.24 ± 0.16	0.49	0.89	1.53
Fructose-6-phosphate	0.16 ± 0.02	0.14 ± 0.02	0.50	0.89	0.12
ChoE (22:5)	0.82 ± 0.06	0.92 ± 0.12	0.50	0.89	0.15
TG 46:2	0.38 ± 0.06	0.46 ± 0.1	0.50	0.89	0.25
tyrosine	0.74 ± 0.09	0.91 ± 0.21	0.50	0.89	0.28
succinic acid	0.68 ± 0.03	0.65 ± 0.02	0.51	0.89	0.03
TG 48:3	0.6 ± 0.21	0.84 ± 0.27	0.51	0.89	0.46
valine	0.93 ± 0.35	1.67 ± 0.93	0.51	0.89	1.00
PC 32:1	0.48 ± 0.14	0.61 ± 0.13	0.51	0.89	0.27
pyruvic acid	13.39 ± 1.64	15.12 ± 1.88	0.52	0.89	0.14
LPC 16:1 e	0.16 ± 0.01	0.16 ± 0	0.53	0.89	0.02
SM 38:1	0.49 ± 0.09	0.56 ± 0.05	0.54	0.90	0.10
ChoE (20:2)	1.16 ± 0.12	1.06 ± 0.09	0.55	0.91	0.07
glycine	2.42 ± 0.32	3.12 ± 1.01	0.56	0.91	0.42
LPC 18:0 e	0.11 ± 0.01	0.1 ± 0.01	0.57	0.91	0.06
PC 40:4	0.22 ± 0.03	0.24 ± 0.04	0.57	0.91	0.17
SM 33:1	0.4 ± 0.03	0.38 ± 0.03	0.58	0.91	0.06
hydroxyproline	0.65 ± 0.11	0.81 ± 0.24	0.58	0.91	0.38
PC 36:2 e	0.01 ± 0	0.02 ± 0	0.59	0.91	0.09
glycerol	3.33 ± 0.22	3.49 ± 0.2	0.60	0.91	0.06

PC 38:5 e	0.1 ± 0.01	0.11 ± 0.01	0.61	0.91	0.10
phenylalanine	0.76 ± 0.09	0.9 ± 0.24	0.62	0.93	0.31
lysine	0.98 ± 0.13	1.1 ± 0.21	0.64	0.93	0.21
PC 36:2	12.51 ± 0.71	13.16 ± 1.08	0.64	0.93	0.09
PC 38:2	0.11 ± 0.02	0.13 ± 0.02	0.65	0.93	0.20
3-hydroxybutiric acid	1.84 ± 0.26	1.7 ± 0.17	0.65	0.93	0.09
SM 36:1	1.3 ± 0.1	1.39 ± 0.15	0.66	0.93	0.11
proline	0.27 ± 0.02	0.29 ± 0.05	0.67	0.93	0.19
LPC 18:2	37.67 ± 2.31	36.37 ± 1.77	0.67	0.93	0.05
methionine	0.12 ± 0.02	0.14 ± 0.03	0.71	0.97	0.20
PC 34:1	4.55 ± 0.61	4.86 ± 0.53	0.72	0.97	0.12
serine	0.3 ± 0.03	0.28 ± 0.04	0.74	0.97	0.13
PC 38:4	18.05 ± 0.86	18.77 ± 1.8	0.75	0.97	0.10
SM 41:1	4.43 ± 0.22	4.32 ± 0.25	0.75	0.97	0.06
Fructose	0.43 ± 0.02	0.44 ± 0.04	0.76	0.97	0.10
SM 42:1	16.98 ± 0.71	16.52 ± 1.2	0.76	0.97	0.07
ChoE (20:4)	80.31 ± 6.86	77.25 ± 6.67	0.76	0.97	0.08
glutamine	1.2 ± 0.24	1.11 ± 0.2	0.78	0.97	0.16
alanine	0.4 ± 0.07	0.45 ± 0.15	0.78	0.97	0.37
beta-alanine	0.08 ± 0.01	0.07 ± 0.02	0.79	0.97	0.28
SM 40:1	4.15 ± 0.29	4.27 ± 0.38	0.79	0.97	0.09
PC 34:3 e	0.02 ± 0	0.02 ± 0	0.80	0.97	0.08
SM 36:2	0.45 ± 0.03	0.47 ± 0.04	0.80	0.97	0.09
histidine	0.16 ± 0.05	0.18 ± 0.05	0.81	0.97	0.33
SM 39:1	0.16 ± 0.02	0.15 ± 0.03	0.81	0.97	0.19
ChoE (17:1)	0.11 ± 0.01	0.12 ± 0.01	0.82	0.97	0.10
PC 33:1	0.09 ± 0.01	0.08 ± 0.01	0.84	0.98	0.08
ChoE (22:4)	5.71 ± 0.53	5.59 ± 0.47	0.87	0.99	0.08
PC 34:1 e	0.13 ± 0.01	0.12 ± 0.01	0.88	0.99	0.08
threonine	1.47 ± 0.19	1.52 ± 0.27	0.88	0.99	0.19
PC 32:2	0.21 ± 0.04	0.21 ± 0.03	0.89	0.99	0.13
asparagine	0.17 ± 0.02	0.17 ± 0.04	0.89	0.99	0.23
ornithine	2.72 ± 0.52	2.86 ± 0.81	0.90	0.99	0.30
LPC 18:1	18.71 ± 1.24	18.91 ± 1.09	0.90	0.99	0.06
aspartic acid	0.51 ± 0.1	0.49 ± 0.11	0.90	0.99	0.21
SM 34:1	19.47 ± 0.89	19.66 ± 1.29	0.90	0.99	0.07
lactic acid	7.09 ± 0.55	7.17 ± 0.43	0.91	0.99	0.06
glutamic acid	0.11 ± 0.02	0.11 ± 0.03	0.95	1.00	0.26
2-hydroxyglutaric	0.69 ± 0.06	0.7 ± 0.04	0.96	1.00	0.06
glycolic acid	3.3 ± 0.21	3.28 ± 0.17	0.96	1.00	0.05
ChoE (22:6)	2.4 ± 0.27	2.38 ± 0.37	0.96	1.00	0.15
PC 30:0	0.05 ± 0	0.05 ± 0.01	0.97	1.00	0.11
alpha-tocopherol	0.91 ± 0.06	0.91 ± 0.08	0.97	1.00	0.09
LPC 18:0	59.41 ± 2.44	59.28 ± 4.04	0.98	1.00	0.07
SM 42:2	13.08 ± 0.92	13.11 ± 0.84	0.98	1.00	0.06
PC 34:0	0.35 ± 0.02	0.35 ± 0.03	0.99	1.00	0.10
PC 36:4	16.73 ± 0.54	16.72 ± 1.17	1.00	1.00	0.07
tryptophan	1.84 ± 0.34	1.84 ± 0.39	1.00	1.00	0.21

Table S7. Plasma feature importance of Random Forest Classifier. The Random Forest Classifier was calculated to sort the most important metabolites in plasma that distinguish between the STD-R and the CAF-R groups. It is shown here only the first 10 metabolites to avoid showing long list. To test it, all metabolites were taken without any filter. DG, diacylglycerol; PC, phosphatidylcholine; LPC, lysophospholipid; SM, sphingomyelin; PE, phosphatidylethanolamine.

Plasma Metabolite	Feature Importance
DG 34:2	0.267
Glyceric acid	0.103
Fumaric acid	0.069
PC 31:0	0.060
DG 34:3	0.034
LPC 16:1 e	0.034
SM 34:2	0.034
SM 32:1	0.034
PE 38:5 e	0.034
DG 36:2	0.034

Table S8. Statistical analysis of urine metabolites in the STD-R and the CAF-R groups. 45 metabolites presented as the mean ± S.E.M. per group shorted by p-value. The summary of univariant analysis is shown including p-value, q-value, FC, right and left chemical shift (ppm); the statistically significant p-values and q-values (< 0.05) are highlighted in bold. 3-HPPA, 3-hydroxyphenylpropionate; HPPA sulfate, hydroxyphenylpropionic acid sulfate; DMA, Dimethylamine; 4-PY, methyl-4-pyridone-5-carboxamide; NAD+, nicotinamide adenine dinucleotide; TMAO, trimethylamine N-oxide; ppm, parts-per-million.

Metabolite	STD-R	CAF-R	*p*-value	*q*-value	FC	Right (ppm)	Left (ppm)
Hippurate	295.91 ± 20.55	145.49 ± 21.45	**<0.01**	**0.01**	0.49	7.531	7.667
o-Coumaric acid	2.16 ± 0.2	6.14 ± 0.76	**<0.01**	**0.04**	2.84	6.529	6.568
3-HPPA	2.31 ± 0.6	21.3 ± 3.91	**<0.01**	**0.04**	9.24	6.793	6.816
HPPA sulfate	1.92 ± 0.32	16.32 ± 3.61	**0.01**	0.08	8.52	2.894	2.920
Tyrosine	7.81 ± 0.5	28.02 ± 6	**0.01**	0.13	3.59	6.857	6.882
Phenylacetylglycine	30.47 ± 1.63	47.65 ± 5.64	**0.02**	0.17	1.56	7.349	7.385
Malate	8.51 ± 1.31	4.8 ± 1.29	0.07	0.43	0.56	2.641	2.653
Citrate	309.65 ± 46.82	214.1 ± 33	0.12	0.66	0.69	2.524	2.583
Fumarate	2.54 ± 0.3	1.88 ± 0.28	0.13	0.66	0.74	6.518	6.531
Sarcosine	4.52 ± 0.31	3.85 ± 0.32	0.16	0.69	0.85	3.593	3.600
N-Acetylglycine	21.66 ± 1.35	19.25 ± 0.9	0.17	0.69	0.89	2.030	2.045
Valine	1.16 ± 0.11	1.43 ± 0.18	0.24	0.84	1.23	0.9878	1.005
Allantoin	200.61 ± 9.28	184.5 ± 10.19	0.27	0.84	0.92	5.379	5.418
Creatinine	155.24 ± 5.74	142.99 ± 8.97	0.28	0.84	0.92	3.036	3.056
N-acetylglycoprteins	50.71 ± 3.06	47.11 ± 1.45	0.32	0.89	0.93	1.997	2.086
1-methylnicotinamide	0.11 ± 0.03	1.4 ± 1.34	0.38	0.91	12.35	9.262	9.288
DMA	40.6 ± 1.94	37.71 ± 2.58	0.39	0.91	0.93	2.718	2.732
2-Oxoglutarate	197.62 ± 41.42	151.94 ± 30.02	0.39	0.91	0.77	2.425	2.460
N.N-Dimethylglycine	10.55 ± 1.67	12.95 ± 2.38	0.43	0.91	1.23	2.929	2.938
Pseudouridine	9.96 ± 0.53	9.29 ± 0.66	0.45	0.91	0.93	7.67	7.684
3-methyl-2-oxovalerate	4.41 ± 0.33	4.79 ± 0.37	0.46	0.91	1.09	1	1.090

Trimethylamine	0.89 ± 0.21	1.09 ± 0.17	0.48	0.91	1.22	2.880	2.895
4-PY	5.17 ± 0.76	4.34 ± 0.89	0.49	0.91	0.84	8.533	8.554
Leucine	13.19 ± 1.05	12.43 ± 0.52	0.53	0.91	0.94	0.9197	0.9517
Acetate	8.37 ± 2.76	6.66 ± 0.82	0.57	0.91	0.80	1.914	1.926
Glycine	7.19 ± 0.63	7.61 ± 0.4	0.59	0.91	1.06	3.564	3.570
Methylamine	4.21 ± 0.39	4.44 ± 0.19	0.60	0.91	1.06	2.606	2.614
Taurine	501.98 ± 67.25	545.59 ± 47.5	0.61	0.91	1.09	3.249	3.290
3-hydroxyisovalerate	4 ± 0.34	4.24 ± 0.33	0.62	0.91	1.06	1.264	1.275
Succinate	57.3 ± 6.5	52.19 ± 7.87	0.63	0.91	0.91	2.397	2.414
2-deoxycytidine	3.41 ± 0.29	3.21 ± 0.39	0.70	0.93	0.94	6.256	6.278
NAD+	0.24 ± 0.04	0.22 ± 0.03	0.70	0.93	0.91	9.355	9.369
Fucose	8.75 ± 0.41	9.06 ± 0.69	0.71	0.93	1.03	1.244	1.264
Alanine	5.07 ± 0.75	4.82 ± 0.33	0.77	0.93	0.95	1.475	1.496
Tryptophan	6.59 ± 0.74	6.34 ± 0.55	0.80	0.93	0.96	7.691	7.720
Betaine	28.95 ± 3.53	27.5 ± 4.22	0.80	0.93	0.95	3.890	3.903
N6-Acetyllysine	23.17 ± 1.7	23.67 ± 1.27	0.82	0.93	1.02	1.979	1.997
Indoxyl Sulphate	6.51 ± 0.69	6.35 ± 0.55	0.86	0.93	0.98	7.693	7.722
TMAO	1.95 ± 0.33	1.85 ± 0.47	0.87	0.93	0.95	3.243	3.248
Formate	6.45 ± 1.4	6.23 ± 0.67	0.89	0.93	0.97	8.450	8.477
α-hydroxyhippurate	0.81 ± 0.13	0.84 ± 0.13	0.89	0.93	1.03	5.515	5.529
Lactate	12.08 ± 1.45	12.04 ± 0.69	0.98	0.99	1.00	1.326	1.348

Table S9. Urine feature importance of Random Forest Classifier. The Random Forest Classifier was calculated to sort the most important metabolites that distinguish between the STD-R and CAF-R groups. It is shown here only the first metabolites to avoid showing long list. To test it, all metabolites were taken without any filter. 3-HPPA, 3-hydroxyphenylpropionate; DMA, Dimethylamine; HPPA sulfate, hydroxyphenylpropionic acid sulfate.

**Urine metabolite**	**Feature importance**
o-Coumaric acid	0.232
3-HPPA	0.196
HPPA sulfate	0.125
Hippurate	0.120
Sarcosine	0.036
Phenylacetylglycine	0.036
DMA	0.018
Tyrosine	0.018
1-methylnicotinamide	0.018

Table S10. Correlation between altered metabolites and alpha diversity. None of the correlations were significative using the correlation test of Spearman. 3-HPPA, 3-hydroxyphenylpropionate; DG 34:2, diacylglycerol 34:2.

		**Metabolites altered**			
		DG 34:2	Hippurate	o-Coumaric acid	3-HPPA
**Alpha diversity**	Shannon	−0.181	−0.181	−0.302	−0.187
	Simpson	−0.176	−0.192	−0.324	−0.187
	Chao1	−0.148	−0.044	−0.451	−0.264
	Observed OTUs	−0.022	−0.192	−0.269	−0.121
	Phylogenetic diversity	0.044	−0.154	−0.126	−0.033

Table S11. Summary of the relation between altered metabolites and genera. The statistical comparisons among metabolites and genus were conducted using test of equal densities; the statistically significant p-values ($p < 0.05$) are highlighted in bold. 3-HPPA, 3-hydroxyphenylpropionate; DG 34:2, diacylglycerol 34:2.

					**Test of equal densities (p-value)**			
**Phylum**	**Class**	**Order**	**Family**	**Genus**	3-HPPA	Hippurate	o-coumaric acid	DG 34:2
Firmicutes	*Clostridia*	*Clostridiales*	*Lachnospiraceae*	-	**<0.01**	0.4	**<0.01**	**<0.01**
Firmicutes	*Clostridia*	*Clostridiales*	*Lachnospiraceae*	*Roseburia*	**0.03**	**<0.01**	0.76	0.65
Bacteroidetes	*Bacteroidia*	*Bacteroidales*	*Porphyromonadaceae*	*Parabacteroides*	0.1	**0.01**	0.48	0.42
Firmicutes	*Clostridia*	*Clostridiales*	*Lachnospiraceae*	*Dorea*	0.12	**<0.01**	0.61	0.71
Firmicutes	*Clostridia*	*Clostridiales*	*Peptostreptococcaceae*	-	**<0.01**	0.68	**<0.01**	0.09
Bacteroidetes	*Bacteroidia*	*Bacteroidales*	*Bacteroidaceae*	*Bacteroides*	0.16	**0.01**	0.94	0.54
Firmicutes	*Clostridia*	*Clostridiales*	*Clostridiaceae*	*SMB53*	**<0.01**	0.89	**<0.01**	0.24
Firmicutes	*Clostridia*	*Clostridiales*	-	-	**<0.01**	**<0.01**	**<0.01**	0.9
Firmicutes	*Clostridia*	*Clostridiales*	*[Mogibacteriaceae]*	-	**<0.01**	**0.02**	0.12	0.5
Firmicutes	*Clostridia*	*Clostridiales*	*Lachnospiraceae*	*[Ruminococcus]*	**<0.01**	0.84	**0.01**	0.06
Tenericutes	*Mollicutes*	*RF39*	-	-	0.32	**0.02**	0.62	0.97
Firmicutes	*Clostridia*	*Clostridiales*	*Lachnospiraceae*	*Coprococcus*	**<0.01**	0.32	**<0.01**	**0.05**
Firmicutes	*Clostridia*	*Clostridiales*	*Ruminococcaceae*	-	**<0.01**	0.4	**0.01**	0.12
Proteobacteria	*Gammaproteobacteria*	*Enterobacteriales*	*Enterobacteriaceae*	-	0.31	0.46	1	0.92
Firmicutes	*Clostridia*	*Clostridiales*	*Peptococcaceae*	*rc4-4*	**<0.01**	0.6	0.17	0.35
Firmicutes	*Clostridia*	*Clostridiales*	*Lachnospiraceae*	*Blautia*	**0.02**	0.61	**0.01**	0.09
Firmicutes	*Clostridia*	*Clostridiales*	*Dehalobacteriaceae*	*Dehalobacterium*	**<0.01**	**0.01**	**0.02**	0.07
Bacteroidetes	*Bacteroidia*	*Bacteroidales*	*S24-7*	-	**0.02**	0.16	0.02	0.33
Firmicutes	*Bacilli*	*Lactobacillales*	*Lactobacillaceae*	*Lactobacillus*	**<0.01**	0.18	**<0.01**	0.2

Firmicutes	*Clostridia*	*Clostridiales*	*Ruminococcaceae*	*Ruminococcus*	**<0.01**	0.21	**0.02**	0.09
Firmicutes	*Clostridia*	*Clostridiales*	*Clostridiaceae*	*Clostridium*	**0.01**	0.15	0.07	0.08
Bacteroidetes	*Bacteroidia*	*Bacteroidales*	*Rikenellaceae*	-	**0.04**	0.59	0.09	0.23
Verrucomicrobia	*Verrucomicrobiae*	*Verrucomicrobiales*	*Verrucomicrobiaceae*	*Akkermansia*	**<0.01**	0.14	0.15	0.07
Firmicutes	*Clostridia*	*Clostridiales*	*Ruminococcaceae*	*Oscillospira*	**<0.01**	**0.01**	**0.01**	**<0.01**
